# When *in vitro* is not enough: *In silico* strategies to investigate functional and dynamical properties of large-scale neuronal assemblies

**DOI:** 10.1063/5.0288838

**Published:** 2025-10-10

**Authors:** Francesca Callegari, Rachele Fabbri, Valerio Barabino, Paolo Massobrio, Chiara Magliaro, Martina Brofiga

**Affiliations:** 1 Department of Informatics, Bioengineering, Robotics, and Systems Engineering (DIBRIS), University of Genova, Genova, Italy; 2 Department of Information Engineering, University of Pisa, Pisa, Italy; 3 National Institute for Nuclear Physics (INFN), Genova, Italy; 4 ScreenNeuroPharm srl, Sanremo, Italy; 5 Neurofacility, Istituto Italiano di Tecnologia (IIT), Genova, Italy

## Abstract

Understanding how neuronal circuits generate complex activity patterns and perform computations remains a significant challenge in neuroscience. *In vitro* neuronal models provide controlled environments to investigate brain microcircuits, their responses to stimuli, and dysfunctions in pathological conditions. While invaluable for direct observation and manipulation, these experiments are also resource-intensive and raise ethical concerns, particularly when involving human-derived neurons. *In silico* models offer a cost-effective, scalable complementary alternative. They integrate multi-scale data, enabling high-throughput investigations and the exploration of mechanisms that may be beyond the reach of experimental methods. These computational approaches support hypothesis generation, data interpretation, and theoretical insight. When combined with *in vitro* studies, they create a synergistic framework that advances our understanding of neuronal function and dysfunction in ways neither method could achieve alone. This review examines computational models developed since 2000 to support *in vitro* neuronal investigations, with a focus on their contributions to understanding network dynamics. This includes topics such as neuronal activity, stem-cell-derived neurons, network topology, and metabolism. We highlight key applications, from predicting mechanisms of neuropathy to exploring network learning and memory. We offer an overview of a corner problem for the development of computational models, that is parameter estimation, and discuss implementation strategies emphasizing accessibility through public repositories. By synthesizing these developments, this review aims to inspire new approaches in computational neuroscience, advancing the study of brain function and dysfunction.

## I. INTRODUCTION

Understanding how circuits of extensively connected neurons give rise to complex patterns of activity and perform computations is still considered one of the unsolved problems in neuroscience.^1^ Despite several research projects on the topic, the mechanisms occurring at the microscale that underlie pivotal high-level processes are still far from being fully understood. In this framework, a great effort is focused on creating “data ladders” that link information available at different scales, providing a clearer picture of how the brain computes.^2^

Several models have been proposed to understand how cells interact in different brain circuits and how these interactions produce different brain functions, ranging from sensory processing to memory consolidation.^3^ Focusing on brain microcircuitry, reliable *in vitro* models serve as controllable tools for investigating circuit dynamics, their responses to external stimuli, and how they may become dysfunctional in pathological conditions. In addition, the functional activity of these circuits can be monitored with several techniques, e.g., patch clamp systems and micro-electrode array (MEA) technology.^4,5^ However, experiments involving *in vitro* neuronal networks are expensive and require proper equipment, sterilized environment, and highly skilled personnel.^6^ Moreover, ethical considerations must also be addressed, especially when embryonic stem cells-derived neurons or cells from patients are involved.

**FIG. 1.**
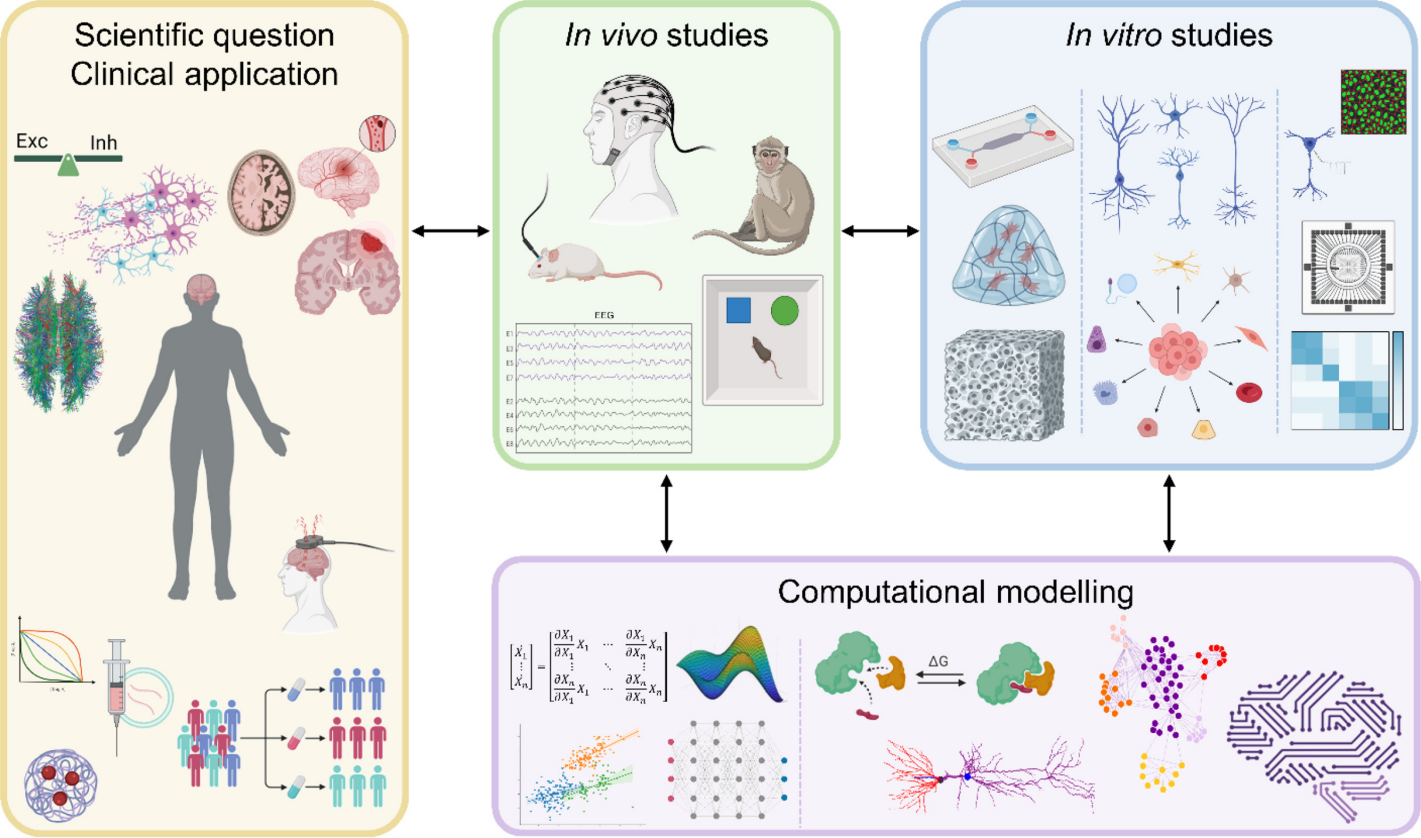
Rationale of network models. The scientific question or clinical application—ranging from understanding the computation, organization, and balance of the brain and their breakdown into pathologies to the development of novel treatments—can be tackled with different complementary modeling techniques, each with their strengths and limitations. *In vivo* studies are the ones that better represent the final target but require non-invasiveness and pose huge ethical questions. *In vitro* models are more controllable, but the right balance between model plausibility and simplicity is under investigation. Computational (*in silico*) models are the farthest from the investigated system as they do not have the intrinsic biological environment that complicates and drives every result, but their setting allows for a more agile analysis of the cause-effect mechanisms within the system, from the micro- to the macroscale. Parts of the figure were created with Biorender.com.

In this scenario, *in silico* models can support experimental investigations by simplifying the study of the mechanisms underlying neuronal function and dysfunction, informing experimental designs, and guiding the interpretation of experimental data. They are usually cheaper, high-throughput, and allow to integrate data on neuronal morphology and activity at different scales, offering the unique opportunity to form hypotheses on mechanisms giving rise to observed activity patterns and verify them computationally when such speculations cannot be inspected *in vitro* (Fig. 1). Furthermore, the current trend in computational neuroscience and the increasing availability of powerful resources has facilitated the development of extensive and accessible model databases [e.g., ModelDB,^7^ EBRAINS (https://www.ebrains.eu/), or the Allen Brain Map (https://portal.brain-map.org/)], which enable neuroscientists to address their scientific questions more efficiently by leveraging existing information.

**FIG. 2.**
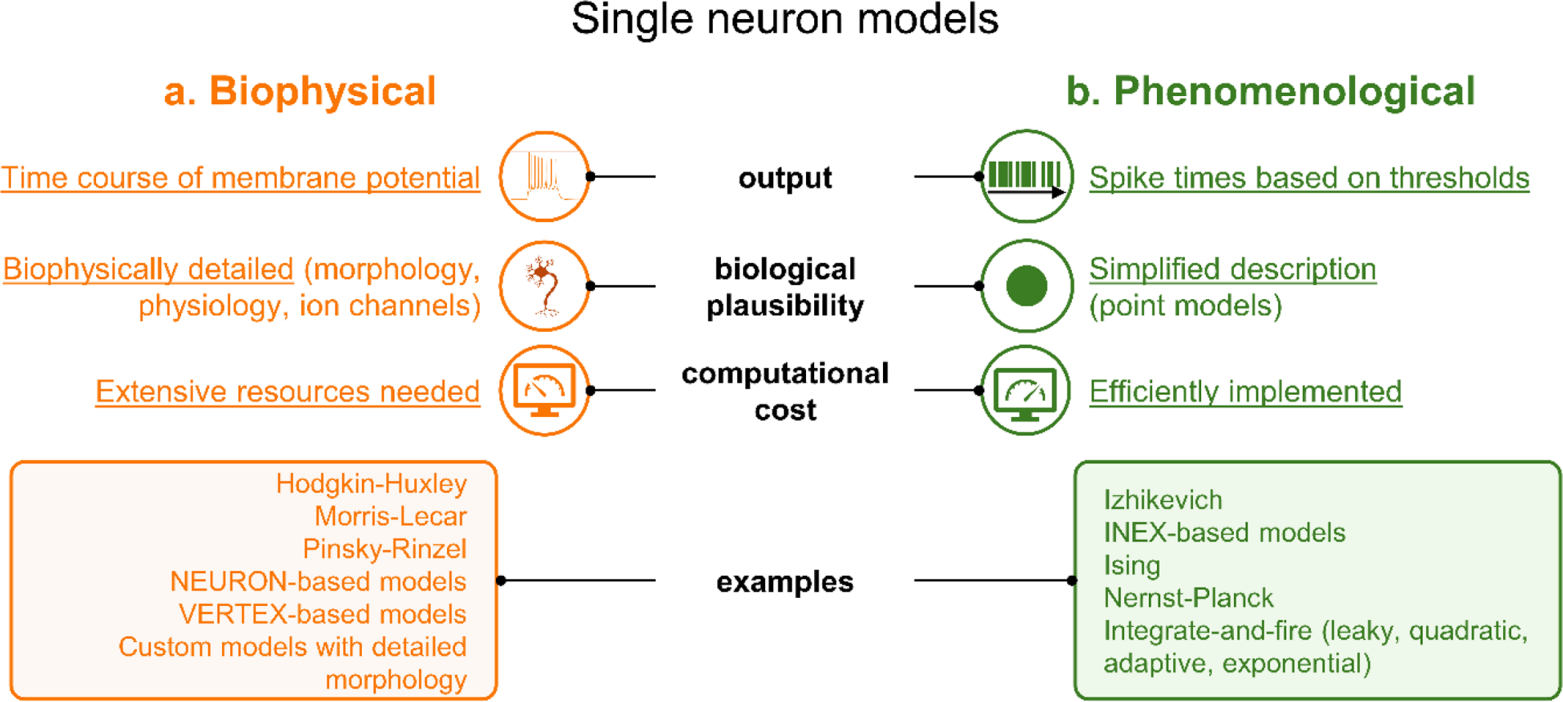
Different strategies to implement single neuron models. (a) Biophysical models capture detailed aspects of the dynamics of the neuron. (b) Phenomenological models use a simplified description of the phenomena.

Different computational approaches can be followed for modeling and investigating network properties. On one hand, biophysical models [Fig. 2(a)] aim at replicating the intricate details of neuronal physiology, including the morphology of individual neurons, the kinetics of ion channels, and the interactions mediated by the synapses. However, faithfully, these models require the setting of a multitude of parameters to capture few selected properties of real neurons. This often requires a great computational cost^8^ and the use of strategies like hand tuning, brute-force search, grid search, simulation-based inference (SBI), and other heuristic approaches.^9,10^

On the other hand, phenomenological spiking models abstract many of the detailed mechanisms of cellular physiology, focusing instead on capturing the emergent properties of neuronal networks [Fig. 2(b)]. They describe neuronal activity using simpler mathematical equations, such as integrate-and-fire models, focusing only on key features of spike generation. For this reason, phenomenological models are computationally more efficient than biophysical ones and are a powerful approach when large-scale population dynamics must be modeled and reproduced.

In light of inspiring new paradigms, approaches, and applications, we provide an overview of the computational models proposed since 2000 to support *in vitro* neuronal network model investigations. We began by analyzing the role of computational models in understanding fundamental network mechanisms that *in vitro* models cannot fully capture. This includes exploring the interplay between neuronal activity (Sec. II A), with particular emphasis on *in silico* models that describe the behavior of neurons derived from human pluripotent stem cells (Sec. II B), network topology (Sec. II C), and metabolism (Sec. II D). We then summarized the primary applications of these models, highlighting their predictive capabilities. This ranges from unraveling cell and network mechanisms associated with specific neuropathies (Sec. III A) to assessing how individual cell properties and connectivity patterns influence the ability of neuronal networks to learn and store information (Sec. III B). In Sec. IV, we present some strategies and algorithms that draw from machine learning and other application fields implemented to infer model parameters from experimental observations. Finally, we outlined the different implementation strategies and technologies used over the years to achieve these goals (Sec. V). To ensure the relevance and rigor of the review, we selected studies using a systematic search of PubMed, Scopus, and Google Scholar. We included articles published from 2000 to 2025 that employed computational models explicitly inspired by or validated against *in vitro* neuronal networks. Keywords for the search included *in silico*, *in vitro*, “neuronal networks,” “computational modeling,” “simulation-based inference,” “machine learning,” “database,” and related terms. Studies focusing exclusively on theoretical or purely *in vivo* models without *in vitro* relevance and on solely theoretical machine learning algorithms were excluded from this review. For the reader's convenience, the articles are organized by year in Table S1. This list includes a brief description of the used *in silico* and *in vitro* models, the objective of the paper as it relates to the scope of this review and in the interest of model sharing and information linkage, a reference to the available repository of the model source code when applicable.

## II. FROM *IN VITRO* TO *IN SILICO*: BUILDING MODELS TO UNDERSTAND NEURONAL NETWORK MECHANISMS

**FIG. 3.**
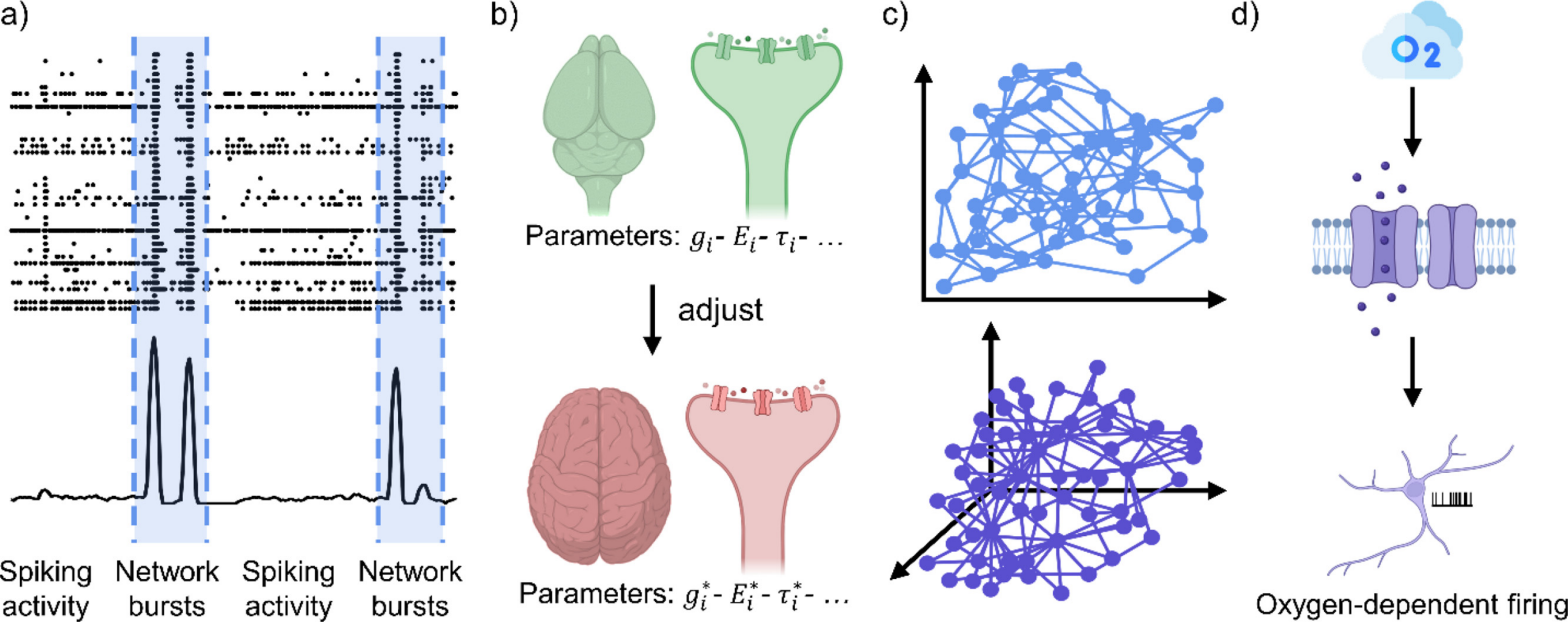
Modeling *in vitro* dynamics with computational numerical simulations. (a) Exemplary raster plot and relative cumulative instantaneous firing rate trace of an *in vitro* rat cortical network, where random spiking and organized network bursts (blue highlight) alternate. *In silico* simulations aim to model and explain the mechanisms behind this trademark. (b) In the framework of translational research, with the advent of neurons derived from human pluripotent stem cells, computational models had to be adapted to replicate their specific electrophysiological behavior, which can differ from that of animal models. (c) From the insurgence of different dynamics and functional observations, numerical simulations try to reconstruct the physical connections in 2D and 3D models to explain *in vitro* behavior, (d) including (hopefully) metabolic constraints, with particular regard to oxygen dynamics since it represents a crucial factor affecting cell functioning and viability. Parts of the figure were created with Biorender.com.

This section examines how computational models enhance *in vitro* studies by revealing essential mechanisms of neuronal networks that are difficult to capture experimentally. These models offer valuable insight into the relationships between cell activity, network organization, and the larger dynamics of neuronal systems, thereby improving our understanding of fundamental physiological and metabolic processes (Fig. 3).

### A. Network dynamics

The analysis of *in vitro* neuronal networks has revealed that their spontaneous dynamics is characterized by slow oscillations, with periods in which the neurons fire in synchrony, thus generating bursts involving the whole network [network bursts, Fig. 3(a)], followed by silent periods with few isolated spikes.^11–13^ This type of activity is considered a sign of the mature formation of the networks *in vitro.*^14^ Understanding the origin of such synchronized bursting activity is still an open research topic and can benefit from computational models.

Overall, the literature proposes different mechanisms for the emergence of network-wide activity. Broadly, these can be categorized into initiation processes driven by specific triggers, such as pacemakers or functional assemblies, or by stochastic fluctuations. In 2010, Gritsun *et al.*^15^ hypothesized the existence of a low percentage (about 4%–16%) of pacemaker neurons characterized by a tonic firing activity in dissociated cortical cultures that acted as network burst triggers. The implementation of this feature in their model, based on Izhikevich neurons [Fig. 2(b)], allowed reproducing the trademark bursting activity, with network bursts characterized by a sharp rise phase and a longer decay phase. Conversely, they contested that this trend was reversed in their purely noise-driven simulations, with rise phases being longer than decay phases. Together with the fact that pacemaker neurons enabled reproducing the flanking phase (i.e., a brief ripple) observed during the decay phase of the network burst, they concluded that a pacemaker trigger better explained the generation of network-wide activity. Another computational explanation of the genesis of these collective population events based on specific triggers was proposed in 2014 by Luccioli *et al.*^16^ They developed a neuronal network model of leaky integrate-and-fire neurons [Fig. 2(b)] to show that small sets of strongly connected neurons—called *functional cliques—*influenced the bursting behavior of the overall network. Thus, both the electrical properties of single-neuron membranes and the pattern of connections enable to replicate *in silico* the synchronized network bursting activity of cultured neuronal networks. Compared to the previous study, in this work, the perspective is shifted from viewing network-wide events as mere consequences of intrinsic neuronal properties to considering them as emergent features of network topology. Within this framework, noise-driven initiation mechanisms gained increasing attention, as will be further discussed in Sec. II C.

The modulation and shaping of collective activity once initiated were largely explored and attributed to both neuronal characteristics, such as variability at the single-cell level, topology, and synaptic plasticity but also to the influence of non-neuronal components, such as glia or extracellular matrix. The examined studies investigated and presented complementary facets of this phenomenon and should not be interpreted as mutually exclusive.

Regarding excitable cells, in 2012, Maheswaranathan *et al.*^17^ observed *in silico* [Izhikevich neurons, Fig. 2(b)] that the periodicity and the strength of spontaneous network bursts were enhanced as cell density, length of synaptic connections, and excitatory to inhibitory ratio increased. One year later, Masquelier *et al.*^18^ pointed out that the inclusion of short-term synaptic facilitation and depression, along with cellular adaptation, in their computational model of leaky integrate-and-fire neurons [Fig. 2(b)] allows generating network bursts with an inter-network burst interval comparable to the one observed *in vitro*. Conversely, in 2004, Persi *et al.*^19^ proposed that inhomogeneity in the values of neurons' membrane resistances, Gaussian noise added to input currents, and dynamic thresholds varying with the firing rate were the key elements to reproduce the profiles of *in vitro* synchronized bursting events in an *in silico* model composed of leaky integrate-and-fire neurons [Fig. 2(b)].

With the increasing knowledge on glia and the mounting evidence of their active role in several brain functions, previously attributed solely to neurons,^20^ researchers started to also investigate the role of astrocytes in network burst regulation. In 2020, Kumar *et al.*^21^ investigated the effects of local glutamate dynamics on synchronous bursting in a network of “Morris-Lecar” neurons [Fig. 2(a)]. They revealed the role of astrocytes in regulating network bursts through glutamate uptake and recycle mechanisms, also providing insight into the processes underlying pathological seizure-like phenomena. A recent computational study by Jiang *et al.*^22^ introduced a scalable framework for modeling tripartite neuron–astrocyte interactions in large-scale spiking networks. Coupling an extended AdEx neuron with Li-Rinzel astrocyte model,^23^ they reproduced experimental findings on astrocyte-driven local synchronization. The model included calcium dynamics within astrocytes and feedback to neurons and was scalable to simulations involving up to a ×10^6^ cells. *In silico* experiments revealed that astrocytes consistently promote synchronization within local neuronal groups, both in asynchronous and bursting regimes. These findings not only replicate known experimental phenomena from *in vitro* and *in vivo* observations but also emphasize how local astrocyte-neuron interactions can shape the emergence of high-order network dynamics, underscoring the significance of glial modeling in understanding functional organization in neural systems. This broader view on non-neuronal contributions to network activity has also been extended to the extracellular matrix, which has recently been investigated as a potential regulator of network bursting by influencing the excitatory–inhibitory balance in neuronal circuits. In 2021, Dzyubenko *et al.*^24^ proposed a synergistic *in vitro* and *in silico* [Fig. 2(b)] investigation that examines the role of the brain's extracellular matrix (ECM) in maintaining the balance between excitation and inhibition (E-I) in neuronal networks. When the ECM was depleted in mature neuronal cultures and in the computational model, there was a selective loss of inhibitory synapses. This loss was accompanied by a compensatory increase in synaptic strength, with a consequent increase in firing and synchronous network activity. They demonstrated that the ECM supports the regulation of the E-I balance—essential for network-wide activity—with inhibitory synapse stabilization, an inhibitory control that was inefficient computationally after ECM depletion.

### B. Dynamics of networks derived from stem cells

In translational research, the development of neurons derived from human pluripotent stem cells (hPSCs) represents a groundbreaking advancement. These neurons serve as human-specific models that allow researchers to study neuronal function and dysfunction directly in the context of human biology. However, the unique electrophysiological properties of hPSC-derived neurons, which are distinct from those observed in animal models, present both opportunities and challenges for computational modeling. To accurately replicate their behavior, computational models must be adapted in several ways [Fig. 3(b)]. Although hPSC-derived neuronal cultures hold great promise for computational modeling, their integration into *in silico* studies remains somewhat limited. This is not due to a lack of experimental work—in fact, numerous studies have characterized various aspects of these networks—but rather to the high specificity of the systems, including differences in differentiation protocols, cell lines, and maturation dynamics. Such specificity makes it difficult to generalize findings across studies, and consequently, computational models capturing these human stem cell-derived networks are still relatively few and often focused on specific features. Nevertheless, these pioneering efforts provide essential insight and establish a foundation for exploring the developmental dynamics, synaptic connectivity, and network activity patterns, such as bursting. They also set the stage for computational modeling aimed at mechanistic interpretation of human *in vitro* dynamics, the investigation of gene- and cell line-specific network properties, the study of neuron–glia interactions, and ultimately for linking cellular and local network phenomena to emergent properties of larger-scale neuronal circuits.

A distinctive feature of neuronal cultures derived from stem cells is that their maturation process closely mimics the early developmental stages of the human brain. More specifically, as neuronal cells mature, they actively seek out connections, extend axons and dendrites, form synapses, and gradually adjust the strength of their synaptic interactions. This synchronized growth of cellular and network-level structures offers a unique opportunity to track the evolution of synaptic connections and burst patterns that are typical of early human brain development. One of the earliest *in silico* models, developed by Lenk's group (2016), used the INEX framework [Fig. 2(b)] l to explore the maturation of human embryonic stem cell-derived neuronal networks,^25^ focusing on the emergence of bursts. By reproducing the emergence of spontaneous spikes and the transition toward structured burst patterns recorded *in vitro* via MEAs, Lenk's model provided a valuable tool for understanding how new synapses shape neuronal dynamics during development.

In parallel, computational models of stem cell-derived neuronal networks have been employed to validate hypotheses on the mechanisms underlying the dynamics recorded *in vitro* as well as to reproduce their response under stimulation. In 2022, Wen *et al.*^26^ adopted an *in silico* model based on exponential leaky integrate-and-fire formalism [Fig. 2(b)], fed with parameters from hPSC-derived cultures, to capture the presence of synchronized bursts when stimulated only at specific frequencies. Computationally, the different response was explained by fatigue and exhaustion mechanisms. These modeling efforts offer an essential bridge between experimental data and mechanistic interpretation.

Importantly, for this field, the advent of induced pluripotency and gene-editing technologies opened new possibilities to explore gene or even individual-specific network mechanisms. This capability has been particularly relevant for disease modeling, enabling the dissection of pathological alterations in connectivity and dynamics. As an example, in 2024, Doorn *et al.*^27^ employed a Hodgkin–Huxley based model [Fig. 2(a)] to investigate the mechanisms influencing the dynamic phenotype of network bursts. They demonstrated that enhanced short-term synaptic depression (STD) alone could reproduce fragmented NBs, a hallmark of disease phenotypes in their MEA recordings. The model successfully isolated the role of STD in disrupting burst structure and highlighted how excitatory persistence—via asynchronous release, NMDA receptors, or short-term facilitation—could further fragment burst activity. Disease mechanisms will be discussed more extensively in Sec. III A.

At the same time, the increasing biological complexity of the *in vitro* systems required the investigation of heterogeneous cellular, such as the role of glial cells in regulating neuronal activity and network bursting. In 2020, Lenk *et al.*^28^ developed a phenomenological model called INEXA [Fig. 2(b)] to study how astrocytes modulate neuronal firing through tripartite synaptic interactions. Simulations demonstrated that astrocytes play a homeostatic role by prolonging neuronal burst durations while preventing network hyperactivity. Moreover, the model showed that astrocytic networks stabilize the firing rate to intermediate levels, independent of baseline neuronal activity, highlighting their ability to regulate neuronal communication dynamically.

Finally, building on the growing complexity, researchers have started to extend the analysis to interactions at the mesoscopic circuit level. In this regard, in 2024, Osaki *et al.* replicated the dynamics of organoid-based models, which recapitulate specific regions of the brain with high fidelity, exploiting the Hodgkin–Huxley formalism [Fig. 2(a)] and incorporating both excitatory and inhibitory neurons.^29^ Their findings illustrate how the structure and connectivity of these networks shape emergent activity patterns, highlighting the importance of studying the interplay between topology and dynamics. Understanding this mutual influence can provide deeper insight into the underlying structures and behaviors of complex systems.

### C. Interplay between dynamics and topology

In Secs. II A and II B, we presented *in silico* models exploited to test hypotheses on mechanisms underlying the synchronized spontaneous bursting activity typically observed in *in vitro* neuronal cultures. We have explored these phenomena in both animal- and human-derived neuronal networks, pointing out that their dynamics may be an emergent feature of network organization. Here, we focus on the role of topological features in shaping their electrophysiological activity. Indeed, over the years, researchers have employed various strategies to investigate how physical constraints, connectivity patterns, and spatial organization influence and govern neuronal network behavior [Fig. 3(c)]. Computational models represent an invaluable support tool in this context, allowing the analysis of structural network properties^30^ and their impact on electrophysiological output,^31–33^ the effects of connectivity changes on spontaneous synchronized activity, and the influence of network parameters, such as synaptic strength, on overall network dynamics.

To illustrate these principles, we now turn to seminal studies that combine *in vitro* recordings and computational modeling to explore how network topology and connectivity give rise to spontaneous bursting activity. Orlandi *et al.*^34^ developed a computational framework with a custom biophysical description of single neurons [Fig. 2(a)] to study the role of network topology in initiating spontaneous coherent neuronal dynamics typically observed in early-stage neuronal cultures. They identified local cascades of activity, or “avalanches,” that either died out or triggered network-wide waves through noise focusing, a mechanism by which specific regions amplify and propagate activity depending on connectivity correlations. Effective networks reconstructed from the dynamics often reveal influential regions that do not correspond directly to the structural network, indicating that functional connectivity arises from the collective dynamics rather than solely from the underlying connectivity. Expanding on this idea, Tibau *et al.* in 2020^35^ investigated how the spatial arrangement of neurons shapes effective connectivity in rat cortical cultures using computational models [quadratic integrate-and-fire, Fig. 2(b)]. By studying cultures with varying degrees of aggregation, they showed that spatial inhomogeneity fosters local connectivity and metric correlations, producing more resilient and coherent networks. Their simulations confirmed that effective connectivity reflects not only network architecture but also the spatiotemporal structure of activity propagation, illustrating how dynamics and spatial organization jointly determine functional network behavior. These spatial and connectivity-dependent dynamics set the stage for the maturation of network activity and the emergence of self-organized criticality,^36^ as explored in developing cortical cultures. In 2010, Tetzlaff *et al.*^37^ exploited mathematical modeling [phenomenological model, Fig. 2(b)] to reproduce the dynamics of maturating cortical networks through neuritic growth, structural overshoot, and pruning under firing-rate homeostasis. They found that networks progressed from sub-critical to supercritical activity before stabilizing in a robust critical state, with ∼20% inhibitory connections being essential to reach SOC, highlighting how excitation–inhibition balance guides physiologically realistic dynamics. Building on the role of inhibition, Massobrio *et al.*^38^ examined how network topology interacts with inhibitory balance to determine SOC and avalanche dynamics. Using Izhikevich-based neuronal networks [Fig. 2(b)] with different topological connectivity, they found that a critical state emerged only in scale-free networks with small-world topologies and physiological inhibition. Random topologies (considered non-physiological conditions) failed to reproduce experimental variability and gave rise only to supercritical dynamical states, emphasizing that SOC arises from the combined influence of inhibitory balance and topology.^35^ Together, these studies indicate that spontaneous bursting and critical dynamics emerge from a multi-layered interplay of structural connectivity, spatial organization, synaptic balance, and developmental maturation. Functional networks reconstructed from activity can reveal effective pathways not evident in the structural network, emphasizing the emergent character of neuronal dynamics. This framework naturally leads to further investigations of more complex architectures, where additional spatial constraints, such as 3D and heterogeneity, further shape network behavior.

Further increasing the level of realism of the considered system, computational approaches can be also useful to elucidate the role of specific interplay among different brain regions. As an example, in the work by Kanagasabapathi *et al.* in 2012, a simple model of two neuronal populations—described via Izhikevich equations [Fig. 2(b)]—was used to evaluate cortical and thalamic dynamics.^39^ By exploring different connectivity schemes between such populations, the study emphasized the role of thalamo-cortical connections in modulating overall network behavior.^40^

With the advent of three-dimensional (3D) constructs, the structure–function relationship is even harder to explore with the available technologies;^41^ computational approaches can truly support its deeper understanding. Three works propose strategies to infer connectivity descriptors of 3D complex structures, based on calcium imaging techniques or MEA recordings. In 2015, Bosi *et al.* derived a network model based on adaptive exponential integrate-and-fire neurons [Fig. 2(b)] to infer architectures able to display results comparable to the biological counterpart in terms of inter-event interval.^42^ In 2021, Bonzanni *et al.* predicted microscale topologies from mesoscale recordings by establishing relations between topological descriptors at different spatial scales, exploiting VERTEX simulator for biophysical neuronal models [Fig. 2(a)]. Their research provided a theoretical framework and a practical tool for studying biological neuronal networks across scales, offering valuable insight into network dynamics and organization.^33,43^ In 2023, Callegari *et al.*^44^ developed 3D *in silico* structures of leaky integrate-and-fire neurons [Fig. 2(b)] by vertically linking 2D networks with different connectivity patterns. They found that a geometrical height of about 200 *μ*m is necessary to induce a change in the firing properties with respect to monolayered networks.

The need to move from 2D to 3D *in vitro* models found its maturity with the advent of organoid technology.^45,46^ Indeed, organoids are highly complex structures that can benefit from computational models. Osaki *et al.* in 2024^29^ developed an *in silico* model of single, fused, and inter-connected organoids exploiting Hodgkin–Huxley formalism [Fig. 2(a)]. By assigning higher synaptic weights to inter-organoid connections, the model accurately reproduced the increased burst frequency observed *in vitro*. This alignment between the model and the experimental results reinforces the idea that robust synaptic connections across organoids drive more intense neuronal activity.

### D. Metabolic-mediated dynamics

Since it is well-known that human brain function strongly depends on the availability of energy, including energetic considerations might allow the implementation of more realistic models of neuronal network dynamics. This is even more crucial for modeling neurons cultured *in vitro.* Indeed, nutrient supply—particularly oxygen, which is the limiting factor in cell culture systems^47,48^—is influenced by environmental and experimental conditions [e.g., the volume of culture medium influencing oxygen availability to the cells in the absence of vascularization, Fig. 3(d)]. This aspect can be useful to model and characterize the functional activity of 3D structures, such as brain organoids, whose inner region is often characterized by a necrotic core due to the lack of oxygenation.^41,49^

In this direction, modifications of both biophysical and phenomenological models to include the metabolic dynamics have been presented over the time, even though few works in the literature consider this aspect. In 2014, Wei *et al.*^50^ modified the Hodgkin-Huxley model [Fig. 2(a)] including the rate at which the membrane Na-K-ATP pumps work depends on the oxygen concentration perceived by the neuron. This model was adapted by Fabbri *et al.* in 2025^51^ to develop a model of the oxygen-dependent firing of neurons cultured *in vitro* that accurately predicts electrophysiological dynamics measured through MEAs [Fig. 2(a)].

With the same strategies, the available models developed for shedding light on the influence of energy demand of neuron activity at the single neuron level (e.g., Ref. 52) up to the brain (e.g., Refs. 53–55) can be adapted and exploited for further evaluating the influence of nutrient supply to network dynamics in both 2D and 3D systems.

## III. FROM *IN SILICO* TO *IN VITRO*: THE PREDICTIVE POWER OF NUMERICAL MODELS IN NEUROSCIENCE APPLICATIONS

This section highlights the predictive power of computational models, proving their capability in helping researchers to guide and refine experimental designs and anticipate experimental outcomes (*in silico* experiments). As a demonstrative example, these models are extremely useful for tuning of scaffold properties, where mechanical and spatial constraints shape the structure and activity of neuronal networks. A notable instance of this approach was demonstrated by Ludl and collaborators in 2020,^56^ who used numerical simulations of Izhikevich neuronal networks [Fig. 2(b)] to predict how the shapes and densities of obstacles influence network architecture and dynamics. Their findings indicated that obstacles facilitated connections at shorter lengths, altered the degree distributions, and enhanced network modularity. These structural changes, in turn, affected the propagation of activity and encouraged the formation of local microcircuits.

**FIG. 4.**
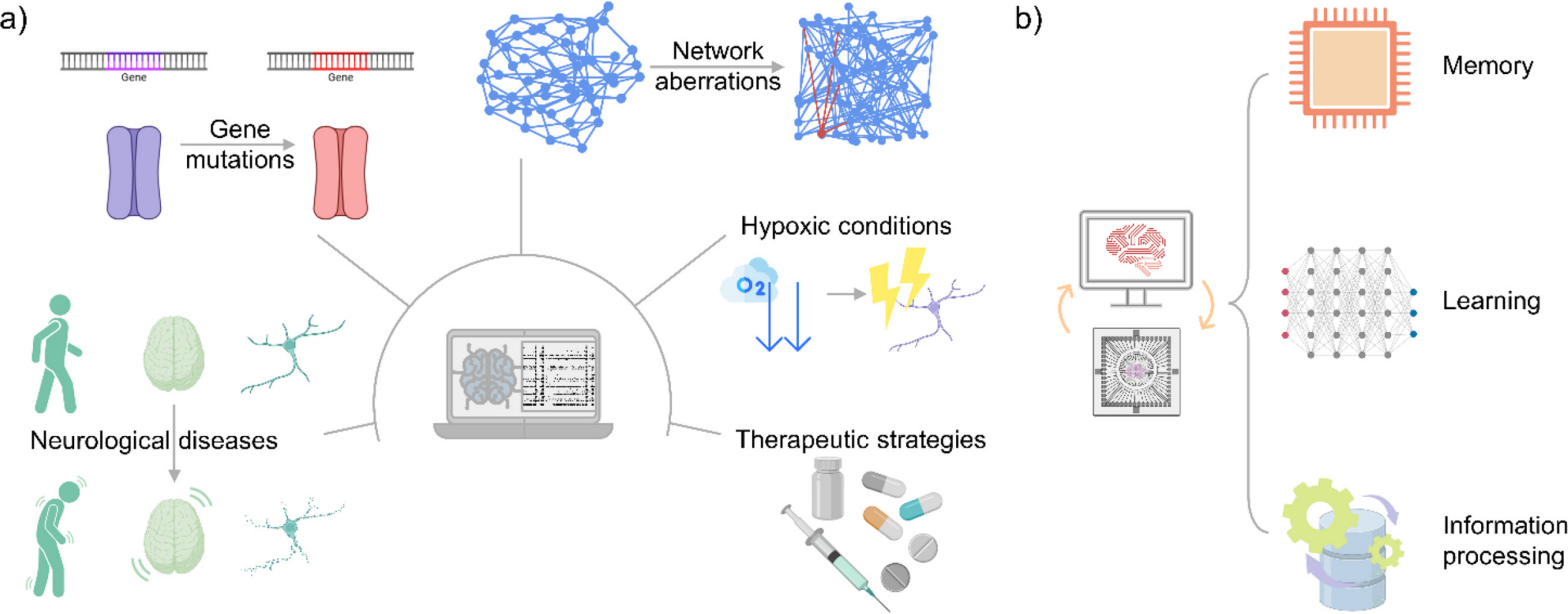
Applications of simulated models of cultured neuronal networks. (a) Computational models can incorporate features associated with diseases and other conditions affecting neurons. This allows for *in silico* predictions regarding the impact of gene mutations, hypoxic conditions, network abnormalities (e.g., node removal), neurological diseases, and the evaluation of therapeutic strategies before conducting *in vitro* experiments. (b) Using computational models of cultured neuronal networks, researchers can analyze cellular mechanisms and topological features that enable higher functions observed in the human brain, such as memory, learning, and information processing. Parts of the figure were created with Biorender.com.

In addition to aiding experimental design, computational models play a crucial role in uncovering neuropathological mechanisms and predicting how cellular and network properties impact learning and memory. These models complement experimental studies by simulating complex neuronal processes, thereby providing deeper insights into the principles of both neuronal function and dysfunction (Fig. 4). Importantly, the research questions addressed in this context span a wide spectrum of applications, with each study focusing on a specific aspect and thus contributing to a heterogeneous body of work. While diverse in focus, these contributions collectively illustrate how *in silico* approaches can anticipate experimental outcomes, refine hypotheses, and inspire new experiments *in vitro*, thus reinforcing their role as a versatile tool in neuroscience research. In Secs. III A and III B, we focus on two main application domains: the study of damage and disease mechanisms and the investigation of higher-order brain functions.

### A. Damage and disease studies

Damage and disease studies in neuroscience have significantly benefited from computational models able to simulate the intricate dynamics of brain networks under different conditions. This approach enabled researchers to explore mechanisms underlying disorders like epilepsy, neurodevelopmental syndromes, and neurodegenerative diseases, while also testing new therapeutic interventions.^57–59^

Damage to the brain's anatomical networks disrupts brain functioning. These disruptions are difficult to predict, depending on the specific location of the injury. As a result, computational neuroscientists have focused their effort on understanding the mechanisms of brain damage, resilience, and robustness.^60–62^ In 2019, Faci-Lázaro *et al.*^63^ used a synthetic Izhikevich model [Fig. 2(b)] in spatially embedded networks inspired by *in vitro* network^34,64^ to simulate damage through targeted and random neuron removal. They found that spatial networks are more resilient than random ones, especially to random removal of nodes and that in both scenarios bursts of activity occurred just before network silencing. The higher resilience in these conditions could be explained by localized subnetworks maintaining high activity, emphasizing the pivotal role of spatial embedding of nodes when analyzing the effects of damage. The study stressed the need to consider the interplay of structure and dynamics (Sec. II B) when assessing resilience to damage in biological neuronal networks.

While targeted damage to neuronal networks provides insight into structural resilience, metabolic disruptions also play a crucial role in understanding how neurological diseases manifest, as suggested by the work of Wei and collaborators.^50^ They developed a model able to replicate seizure-like events by inducing hypoxia, leading to an ion imbalance in the extracellular environment responsible for heightened excitability and the occurrence of spontaneous seizures. Finally, they demonstrated how variations in tissue architecture (cell density, depth, and glial distribution) influence the hypoxic state and, consequently, neuronal behavior, proposing several mechanisms for seizure occurrence. On the topic, Stasenko *et al.*^65^ have explored how astrocytic dysfunction—such as that caused by SARS-CoV-2 infection—can impair the emergence of coherent brain rhythms in neuronal networks. The authors developed a spiking neuronal network model based on Izhikevich equations [Fig. 2(b)] which interacts with astrocytes via glutamate-mediated feedback. The model incorporated virus-induced reductions in gliotransmitter release, resulting in a range of network-level dysfunctions, from intermittent desynchronization to complete loss of rhythmic bursting. The authors provided a mathematical framework for understanding the impact of the coronavirus on infected astrocytes and metabolism, which may be the cause of some of the post-COVID pathological states.

The power of computational models has also proven essential in validating therapeutic strategies before conducting experiments. In 2021, Trujillo *et al.*^57^ developed computational models to gain a deeper understanding of disease mechanisms driven by MeCP2 gene mutations. While synaptic aberrations are hallmarks of MeCP2 deficiency,^66–68^ compensatory mechanisms can influence network function. To understand whether addressing synaptic dysfunction will improve neuronal activity, Trujillo *et al.* used phenomenological recurrent neuronal networks [Fig. 2(b)] to simulate synaptic defects in MECP2-knockout neurons and predict changes in network dynamics following synaptic restoration. The model showed that rescuing synaptic structure could significantly increase neuronal activity across various network configurations, allowing the exploration of complex interactions between synaptic structures and network function. This strategy has proved to be useful to guide drug selection and, thus, aid the development of treatments for restoring network dynamics when MeCP2 gene mutations occur, e.g., in patients affected from Rett syndrome. The same mutation in Rett syndrome was also studied using a different approach in 2022 by Mok *et al.*^69^ By combining protein expression analysis, neuronal morphology, electrophysiology, and MEA recordings, they were able to capture a wide spectrum of RTT-associated phenotypes across multiple levels. The *in silico* model, specifically an adaptive leaky integrate-and-fire neurons [Fig. 2(b)], helped elucidating how changes in Na^+^ and K^+^ currents in individual neurons could drive the observed differences in the network burst frequency.

Similarly, computational models have been applied to study other severe neurological disorders, providing key insight into the role of sodium channel mutations. In 2023, Doorn *et al.*^58^ combined neuronal networks derived by induced pluripotent stem cells and *in silico* models to study Dravet syndrome, a severe infantile epileptic encephalopathy, caused by mutations in SCN1A.^70–72^ Since excitatory networks derived from Dravet syndrome patients exhibited a distinct phenotype on MEAs, it was hypothesized that this phenomenon was caused by alterations in sodium channel dynamics linked to the SCN1A mutation. Consequently, researchers developed a biophysical model—namely, a revision of the Hodgkin–Huxley model [Fig. 2(a)]—showing that modifications to sodium channels alone could not account for all the observed functional phenotypical changes observed *in vitro*, but rather a change in network burst rate was only achievable. A year later, they used the same *in silico* framework to predict mechanisms at play for the emergence of the dynamic phenotype observed in different syndromes, such as Dravet, GEFS+, Kabuki, and Rett.^27^ The electrophysiological hallmarks they observed in these pathologies are fragmented NBs, essentially irregular and intermittent spiking within a burst period. They reproduced the fragmented NBs and identified a possible mechanism to explain the emergence of this behavior (Sec. II A). They validated their hypothesis by increasing STD pharmacologically in healthy cultures, supporting the predictive relevance of their computational model. Furthermore, the *in silico* model revealed that manipulating persistent excitation and adaptive mechanisms could act on NB fragmentation. These results offer mechanistic hypotheses that may generalize across diverse patient-derived models and identify potential therapeutic targets modulating STD or persistent excitation pathways. In 2025, the researchers employed their model to investigate the biophysical mechanisms underlying disease-specific activity patterns by using a simulation-based inference (SBI) algorithm^73^ (cf. Sec. IV). The authors showed that SBI could accurately recover ground-truth parameters from synthetic data and estimate plausible parameter distributions that reproduced the electrophysiological activity of both healthy and patient-derived cultures. The method also identified molecular pathways modulated by pharmacological treatments, highlighting how disease mechanisms could be disentangled in a data-driven, systematic manner. Rather than relying solely on *a priori* hypotheses, this approach allows for a comprehensive exploration of candidate mechanisms that are consistent with experimental data, thereby guiding more targeted experimental follow-ups. In the context of iPSC-based *in vitro* models, which are often constrained by biological variability and experimental throughput, this study illustrates the potential of SBI to enhance mechanistic interpretability and support translational research. In 2022, Kress *et al.*^74^ exploited the Hodgkin–Huxley model [Fig. 2(a)] with parameters obtained from previous studies using neurons derived from pluripotent stem cells, induced from healthy subjects or carrying specific sodium channel mutations linked to epilepsy. From the model, several electrophysiological features defining network connectivity, activity, waveform shape, and complexity were extracted. Finally, different machine learning algorithms were tested, demonstrating their ability to predict under which genetic category the subject falls.

Studying conditions like epilepsy also requires examining the role of the balance between excitatory and inhibitory neurons in both healthy and pathological brain networks,^75,76^ providing valuable insight into the fundamental principles governing network activity. An imbalance in excitation and inhibition within local neural circuits was studied *in vitro* and *in silico* in another pathology, schizophrenia, where it is supposed to constitute a central mechanism of the disease. Dzyubenko *et al.*^77^ employed a combined approach integrating synapse-specific quantification, spontaneous activity recordings from MEAs, and computational modeling [with Izhikevich formalism, Fig. 2(b)] to assess the effects of first- and second-generation antipsychotic drugs on hippocampal neuronal cultures. By simulating synaptic rearrangements observed after drug treatment, the *in silico* model accurately reproduced experimental patterns of network activity. Notably, the second-generation antipsychotic increased synchronization and bursting activity more than the other drug, suggesting a mechanistic link between drug-induced connectivity changes and their cognitive effects. Like the work of Trujillo *et al.* or Doorn *et al.*, this study highlights the potential of hybrid experimental–computational approaches to discover therapeutic approaches by revealing how pharmacological treatments reshape network-level dynamics in neurological and psychiatric disorders.

### B. Modeling the mechanisms for higher brain functions

By modeling the spatiotemporal activity of neuronal networks and integrating experimental data, *in silico* approaches enable to explore fundamental mechanisms underlying cognitive processes in a controlled environment, providing valuable insight into how the brain encodes, stores, and retrieves information.

Recent empirical studies using large-scale recordings of neuron populations, including *in vitro* experiments, have explored the relationship between memory states and spike-timing patterns, highlighting the need to capture both reliable firing patterns and their precise timing. In 2009, Liu and She^78^ used a recurrent neuronal network based on integrate-and-fire formalism [Fig. 2(b)] to model the dynamics of memory operating on two timescales, to account for both neuronal and synaptic rapid variations as well as homeostatic presynaptic-dependent scaling. They found that network topology plays a significant role in learning. Locally connected networks have been shown to embed more memory states, suggesting that the structure of neuronal connections directly impacts the system's ability to retain and recall information. Understanding the mechanisms of learning, particularly of reinforcement learning, was also the aim of Kim *et al.*,^79^ who, in 2019, investigated the role of striatal cholinergic interneurons in regulating dopamine and facilitating reinforcement learning within the striatum via computational modeling. The model captured how the pause in interneuron activity, which occurs after excitatory stimuli, creates a window for dopamine release, which, in turn, modulates the learning processes by encoding reward prediction errors. The alterations in interneuron–dopamine relationship can impact learning performance, particularly under conditions of dopamine deficiency, as seen in Parkinson's disease. Thus, the work also provided potential applications in clinical settings.

Regarding higher brain functions, in 2012, Maheswaranathan *et al.*^17^ developed an *in silico* model of cortical neurons (Sec. II A), relating various features of the network—such as cell densities, small-world connections, and an excitatory-to-inhibitory ratio—to effective information processing. In 2015, Ju *et al.*^80^ proposed a spiking recurrent network model based on LIF formalism [Fig. 2(b)] with short-term synaptic plasticity to explain how disordered cortical cultures can classify complex spatiotemporal inputs, such as short musical sequences. The model predicted that memory retention and stimulus classification rely on the interplay between recurrent connectivity and short-term synaptic plasticity, without requiring NMDA-mediated plasticity.

These insights gained through computational models pave the way to systematically explore memory, learning, and information processing mechanisms.

## IV. FROM OPTIMIZATION TO SIMULATION BASED INFERENCE: PARAMETER ESTIMATION IN COMPUTATIONAL NEUROSCIENCE

Inferring the parameters of computational models from experimental data are a fundamental challenge in neuroscience. This challenge is common to all the modeling strategies reviewed here, from mechanistic models investigating the emergence of network dynamics to models tailored to reproduce pathological behaviors. Regardless of the application domain, identifying parameters that enable models to reproduce key experimental features is essential for interpretability and predictive power. Traditionally, parameter estimation often begins with hand tuning, i.e., a manual adjustment of model parameters based on expert knowledge and trial-and-error procedures. Despite its simplicity, this approach is still commonly used, particularly when computational resources are limited or when the model structure is relatively simple. However, hand tuning is subjective, not scalable, and does not offer any assessment of parameter uncertainty.

To overcome these limitations, a range of computational strategies has been developed. These include traditional optimization methods like genetic algorithms or grid searches, which search for the single best-fitting parameter set and more recent simulation-based inference (SBI) techniques.

Parameter optimization methods frame the inference problem as a global search for the set of model parameters that minimizes some distance between the model's behavior and experimental observations. A widely used tool in this domain is BluePyOpt an open-source Python library developed within the Blue Brain Project by Van Geit *et al.* in 2016.^81^ BluePyOpt offers a modular interface that allows users to plug in custom models ranging from simple equations to multi-compartmental simulations interfaced through Python. It integrates several key libraries: DEAP (a mature library for evolutionary algorithms), parallel computing libraries, and data analysis tools, such as eFEL (Electrophysiology Feature Extraction Library). To perform an optimization, the user must define an evaluation function that maps model parameters to a fitness score, often via simulation. BluePyOpt can then explore the parameter space to find optimal settings, particularly for complex biophysical neuron models, such as multicompartment Hodgkin–Huxley cells or for synaptic plasticity models. Despite their success, these optimization approaches have notable limitations. They are often computationally expensive, provide only point estimates of the parameters, and offer limited insight into uncertainty.

Recently, SBI has gained traction in computational neuroscience. SBI represents a paradigm shift by aiming to infer the full posterior distribution over parameters rather than just point estimates. These methods leverage advances in machine learning [e.g., using recurrent neural networks (RNNs)] and probabilistic modeling to learn complex mappings between parameters and observations, even in systems with intractable likelihoods. SBI encompasses a growing set of techniques designed to perform inference on complex models whose likelihood is intractable or unavailable, making them particularly suited for complex dynamical systems in neuroscience. Recent advances in machine learning, probabilistic programming, and differentiable programming have broadened the scope and power of these approaches, enabling efficient inference over high-dimensional parameter spaces and complex model behaviors. A variety of algorithmic approaches have been proposed, each addressing different aspects of the inference problem, as reviewed by Cranmer *et al.* in 2020^9^ and Lueckmann *et al.* in 2021^82^ who proposed a benchmark framework. Below, we present an overview of key approaches.

A powerful approach named sequential neural posterior estimation (SNPE) was presented by Lueckmann *et al.* in 2017^83^ and Gonçalves *et al.* in 2020.^84^ SNPE is a likelihood-free inference approach based on training a neural density estimator to approximate the posterior distribution over model parameters. SNPE leverages deep neural networks trained on synthetic data to estimate the full posterior distribution of parameters compatible with experimental observations or selected summary features. The network is trained over multiple rounds of adaptively chosen simulations, allowing efficient exploration of the parameter space. This allows flexible and scalable parameter identification without requiring explicit likelihood functions. The authors validated the algorithm on synthetic data and applied to real *in vitro* recordings. Although demonstrated here for single-neuron models, the approach is general and applicable to population-level models as well.

Building on SNPE, in 2019, Greenberg *et al.* presented the automatic posterior transformation (APT) approach.^85^ APT combines the benefits of posterior-targeting approaches with the flexibility typical of likelihood-based methods. Using recurrent neural networks, it operates directly on raw, high-dimensional time-series data, such as multi-neuron spike trains to map the full observation to a posterior estimate, thus bypassing the need for summary statistic engineering. This makes APT particularly appealing for neuroscience applications where temporal structure is rich and data contains large fractions of uninformative features.

In 2019, Papamakarios *et al.*^86^ proposed a different approach, which they named sequential neural likelihood (SNL). It is a simulation-based Bayesian inference method that learns an explicit model of the likelihood using a conditional density estimator based on autoregressive flows instead of estimating the posterior directly. This allows the use of standard inference algorithms, such as Markov chain Monte Carlo (MCMC), to draw the posterior estimates. SNL is trained sequentially: after each training round, an MCMC sampler proposes new simulation parameters based on the current likelihood model, focusing simulation effort on high posterior density regions. This targeted simulation strategy dramatically reduces the number of simulations required and mitigates the proposal bias, making the method highly efficient.

Moving further, in 2020, Hermans and his group proposed a different model to tackle intractable likelihoods.^87^ Their algorithm, Amortized Approximate Likelihood Ratio MCMC (AALR-MCMC), replaces traditional likelihood evaluations in MCMC with an amortized likelihood-to-evidence ratio estimator. Rather than relying on repeated simulator calls during inference, this setup uses a neural network trained offline to map mapping estimate likelihoods from observations and parameters. Once trained, the amortized estimator enables posterior sampling without requiring further simulation, a major computational advantage in neuroscience contexts where simulators are costly and complex. Importantly, as for APT, AALR-MCMC operates without the need for summary statistics, learning directly from high-dimensional data.

Finally, in 2021, Bittner and collaborators reframed the SBI problem by focusing on emergent computations or functional properties rather than raw data fit.^88^ Their Emergent Property Inference (EPI) approach uses deep neural networks to learn parameter distributions that give rise to specific emergent computational properties, such as neural response variability or circuit-level amplification, bridging the gap between abstract theoretical models and empirical observations. This holds particularly when the goal is to constrain mechanistic hypotheses to reproduce defined computational outcomes. In fact, EPI constrains the inference process to match high-level statistics that reflect the computational role of a circuit, overcoming the problem of confounding irrelevant variability.

The adoption of SBI methods in neuroscience has been increasing, although these methods were originally developed within the broader context of Bayesian statistics and simulation-based modeling. Many of the presented algorithms were not explicitly designed for neuroscience applications; however, their growing use in this field demonstrate their applicability to complex dynamical systems, such as neuronal networks. Despite their promise, SBI methods face important limitations, especially within this new field of application due to the high-dimensional and noisy nature of biological data. These include the curse of dimensionality with limited simulation budgets, weak parameter identifiability, sensitivity to misspecified or overly compressed summary features, and potential failures when experimental data deviate from simulated training distributions.^9^ Moreover, amortized neural density estimators can exhibit “amortization gaps,” leading to biased posteriors. The reviewed papers proposed different mitigating strategies as sequential experimental design,^86^ automatic posterior transformation,^85^ dimensionally reduction guided by biophysical priors,^84^ the use of informative priors or regularization, and systematic posterior diagnostics, such as simulation-based calibration.^89^ While these challenges remain, the ongoing work is making SBI increasingly robust and scalable for neuroscience applications. In this review, we discussed synergistic *in silico*-*in vitro* approaches to address numerous scientific questions. In this specific context, one example of SBI applied to neuroscience is the work by Doorn *et al.*^73^ already presented in Sec. III A. They exploited a python package implementing SBI^90^ to infer the parameters that give rise to specific physiological and pathological phenotypes observed in electrophysiological data.

## V. FROM THEORY TO PRACTICE: IMPLEMENTATION APPROACHES AND TECHNICAL STRATEGIES

**FIG. 5.**
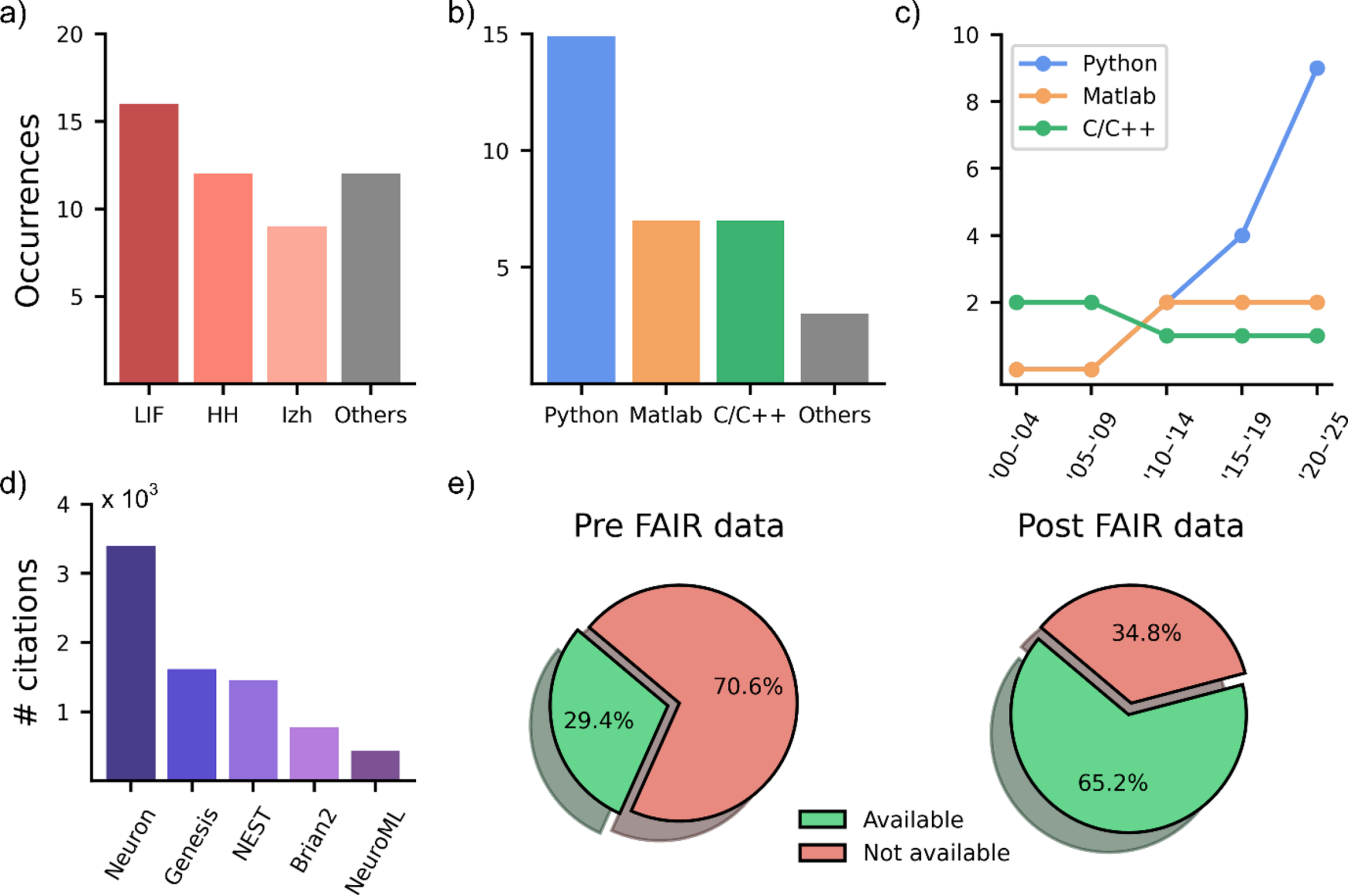
Implementation strategies. (a) Neuronal models adopted in the reviewed papers. (b) Coding language in the reviewed papers and (c) their trend over the years. (d) Number of citations of simulation environments on Google Scholar. (e) Data availability before (left) and after (right) the introduction of FAIR principles in 2016.

The choice of the simulation environment significantly influences the type of neuronal model used for the specific application (Fig. 2). This decision often requires balancing a high biological realism with reduced computational costs. In this review, 94% of the discussed works uses single-compartment models. Most of these models employ simpler phenomenological approaches (about 51%), such as leaky integrate-and-fire and Izhikevich neurons, rather than more complex conductance-based models like Hodgkin–Huxley (about 24%) [Fig. 5(a)]. It is worth noticing that a powerful application of *in vitro* neuronal models is to directly test pathologies (such as channelopathies) or drugs that target specific channels. This highlights the significance of using models like Hodgkin–Huxley, which capture such dynamic processes, even if they require more computational resources. Thanks to the emergence of neuroinformatic platforms providing high-performance computing resources, such as EBRAINS (https://www.ebrains.eu/)—a European initiative closely associated with the Human Brain Project^91^—and NetPyNE,^92^ the computational challenge posed by biophysically detailed models is mitigated. In 2025, simulating large networks of thousands or millions of Hodgkin–Huxley neurons on these platforms, traditionally running for days or weeks, requires only minutes or hours. Accessing such platforms enables the use of extensive databases of both models and data. However, while these platforms substantially lower the technical barrier, their scalability to mesoscale systems and organoids, which represent the most complex models within the scope of this review, remains challenging. In contrast to *in vivo* models, where atlases and curated databases facilitate mesoscale network construction, organoid systems lack systematic characterization, slowing down model design and reducing the reliability of simulations. Indeed, tools such as the Brain Scaffold Builder (https://github.com/dbbs-lab/bsb^93^) illustrate how detailed structural information can accelerate model construction, but equivalent resources remain scarce for organoid-level studies. Indeed, similar to *in vivo* systems, organoids introduce additional complexity, including evolving cell-type heterogeneity, layered 3D architectures, stochastic long-range projections, and metabolic gradients with respect to other *in vitro* structures. However, while significant progress has been made, their experimental characterization remains less established. Current *in silico* approaches typically address these features only in isolation—for instance, 3D connectivity,^44^ or simplified reaction-diffusion models for oxygen availability.^51^ Also, groups who focus on organoid studies have used simplified models that reproduce few selected features: Trujillo *et al.*^57^ employed reduced network representations as predictive tools to generate testable hypotheses, and Osaki *et al.*^29^ modeled fused and connected organoids using simplified neuronal networks with optimized synaptic weights to capture inter-organoid bursting activity. These approaches demonstrate that simplified models can yield mechanistic insights and hold predictive value, but they remain partial representations of organoid dynamics. A key open challenge is to integrate these features within a single tractable model. Hybrid strategies that combine heterogeneous point-neuron networks with mesoscale conduction-delay maps and coarse metabolic surrogates may offer a pragmatic path forward, while advances in high-performance simulators will be critical to approximate realistic organoid behavior.

This process may be helped by the growing culture of sharing data and models that is spreading in the neuroscientific community, together with the diffusion of new versatile and open-source programming languages. From the analyzed literature, we observed that Python-based simulations have gained popularity over the years [Figs. 5(b) and 5(c)]. The advantages of Python include its simplicity, versatility, and open-source nature, which allows researchers to easily access codes and models. This phenomenon may elucidate why, among the five most frequently cited simulators on Google Scholar [Fig. 5(d)], three offer full compatibility with Python (NEURON, NEST, and Brian2), while the remaining two offer indirect compatibility (GENESIS and NeuroML).

Neuronal simulators, such as NEURON,^94^ GENESIS,^95^ NEST,^96^ and Brian2,^97^ are widely used to model the nervous system across various scales and applications [Fig. 5(d)]. These simulators address the trade-off between performance and flexibility by implementing code generation. This method automatically translates high-level user-defined models (typically written in Python) into optimized low-level code (such as C/C++), which is then compiled and executed as the backend. This approach ensures high performance while maintaining flexibility and extensibility, making it easier to support new architectures (like GPUs) and add custom functionality. Additionally, tools like PyNN^98^ provide a simulator-independent API that combines Python's expressive capabilities with automatic code generation, facilitating efficient and flexible simulation workflows.

Finally, it is worth mentioning the relevance of the outcome of the simulations in terms of reproducibility and usability. The FAIR principles,^99^ introduced in 2016, pushed a noticeable shift in terms of code availability (i.e., models and data) [Fig. 5(e)]. In fact, data availability has risen from 29.4% to 65.2%. Access to models and data are crucial for accelerating progress in the neuroscientific community.

## VI. CONCLUSION

In this review, we provided a critical overview of studies published since 2000 where computational models have been exploited to support experimental investigations performed with *in vitro* neuronal networks. Together, the two modeling approaches—*in vitro* and *in silico—*create a synergistic framework: computational simulations precisely control variables and environmental conditions, while *in vitro* models offer essential biological data to validate and refine these computational predictions. Most research efforts have focused on developing models to understand the mechanisms responsible for the synchronized bursting patterns observed in cultured dissociated neurons—both in animal and human cells—and to assess how topological structures influence electrophysiological outcomes. Moreover, by incorporating metabolic considerations into the models of cultured neuronal networks, their physiological relevance has been increased, capturing with more detail the dynamics observed experimentally. Computational models are increasingly being used to predict the outcomes of experiments, effectively creating an *in silico* laboratory. In this context, researchers have modeled disease-related abnormalities in neuronal networks, allowing for a computational assessment of their impact on electrical activity. This approach holds promise for evaluating various therapeutic strategies, providing significant gain in throughput as well as savings in time and money. Furthermore, computational models have been employed to explore the cellular foundations of memory, learning, and information processing.

The effectiveness of this approach has been proven by researchers who have predicted how different treatments might affect patients by integrating stem cell technology and computational modeling of disease processes, paving the way for personalized medicine and more effective interventions. However, most of the analyzed works have focused on neuronal models of diseases that involve only single-gene mutations, e.g., MeCP2^57^ or SCN1A.^58^ Realistically, diseases often involve more complex genetic landscapes. While in principle computational approaches could be extended to capture polygenic or multifactorial disease mechanisms, this would move significantly beyond the level of complexity typically addressed by the corresponding *in vitro* systems. As such, future perspectives may consider more integrative models that account for diverse biological disruptions, but to remain practically useful, *in silico* frameworks should stay closely connected to experimentally tractable systems. In this sense, computational models are best employed as predictive tools that generate falsifiable hypotheses and mechanistic insights, bridging detailed data with interpretable theories of brain function and ultimately supporting experimental design.

Higher brain functions emerge from complex and interconnected cerebral regions, composed of different cell types that interact to convey information. Despite the effort to effectively correlate the connectivity among cells, their metabolic activity, and their emerging dynamics, few computational studies have provided a comprehensive and reliable assessment of the role of how the interaction of heterogeneous cells—such as those in the cortical-hippocampal or cortical-thalamic-striatal circuits—contribute to brain function. To accomplish this feat, it is necessary to match the “activity phenotype” of the different types of neurons to verify whether their isolated and interconnected behavior mirrors the changes observed *in vitro*. In this context, it is important to extend the focus beyond neuronal populations and include glial cells. In addition to astrocytes, whose role in regulating network dynamics through tripartite synapse was discussed (Secs. II A and II B), it should be noted that other glial populations also play crucial roles in shaping neuronal network activity. Microglia actively regulate synaptic pruning and immune signaling,^100^ thereby influencing connectivity and excitability during both development and disease. Similarly, oligodendrocytes control axonal conduction velocity through activity-dependent myelination, a process that modulates oscillation frequency and long-range coherence.^101^ Though a few computational studies have attempted to incorporate these mechanisms, the available literature remains limited. For example, one recent modeling study investigates microglial contributions at the whole-brain level,^102^ but this approach lies beyond the primary scope of our review. Nonetheless, readers interested in the broader context of glial modeling can refer to dedicated reviews,^20,103,104^ which provide an overview of emerging strategies to incorporate astrocytic and glial functions into computational frameworks. However, including these additional cellular mechanisms *in silico* may require implementing and calibrating an increasing number of parameters, which poses significant computational challenges. It also calls for the development of new solutions: machine learning algorithms (e.g., Ref. 84) are particularly suited for exploring complex parameter spaces, identifying their optimal configurations. In this context, simulation-based inference is a powerful paradigm to discover model parameters by targeting their full posterior distributions, allowing models to remain flexible yet constrained by empirical data (Sec. IV). These approaches, often rooted in machine learning and probabilistic inference, show particular promise in neuroscience. Coupled with extensive databases of experimental data and models (e.g., Ref. 81), these approaches enable more systematic exploration and optimization of activity dynamics.

To ensure that the computational models used in a predictive way (Sec. III) replicate and infer biologically plausible behavior, experimental data must play a central role in validation. In practice, however, validation of *in silico* models against *in vitro* data has so far been approached in a heterogeneous manner. Common strategies include qualitative comparisons (e.g., raster plots of neuronal spiking activity or calcium dynamics in astrocytes), task-related performance measures, or hypothesis testing, where predictions generated *in silico* are subsequently verified experimentally. More quantitative approaches rely on specific metrics tailored to the phenomenon under investigation, such as firing rates, bursting and network bursting statistics, spectral analyses, or calcium signaling. However, these metrics are not standardized across studies, and systematic benchmarking is still rare. While only a minority of works explicitly frame their discussion in terms of model uncertainty of benchmarking,^65^ many do acknowledge the limitations of their models and clearly state the assumptions on which they rely, clarifying which aspects may constrain generalizability. Overall, the field would benefit from shared validation standards and the use of collaborative platforms (e.g., EBRAINS, ModelDB, Sec. V), which could facilitate reproducibility, comparability, and more quantitative integration of *in silico* and *in vitro* approaches. This applies, for example, to the development of models that accounts for metabolism: oxygen consumption measurements could be performed while recording electrophysiological data via MEAs. Such multi-modal datasets not only refine the models but also enhance their reliability by linking physiological processes with observed behaviors.

Using *in silico* models to enhance our knowledge about the neuronal functioning of *in vitro* networks and field-related challenges can be facilitated by employing novel computational platforms. Recent advancements in neuroinformatics have led to the development of novel infrastructures that provide access to high-performance computing resources and a comprehensive set of tools for simulating, analyzing, and understanding brain-like networks. These platforms allow their inspection at various levels of abstraction, from cell structures to large-scale brain models. Advanced neuronal simulation platforms have notably reduced the technical barriers for neuroscientists, equipping them with powerful tools to simulate neuronal activity. This allows researchers to focus on biological and with user-friendly interfaces, extensive libraries, and modular design support, neuroscientists can now construct and simulate complex neuronal models without requiring advanced programming or computer science expertise.

Finally, it is worth highlighting that a new generation of approaches is emerging that frames neural processes explicitly as dynamical systems, often by leveraging machine learning models, such as recurrent neural networks (RNNs). These models aim to replicate the complex, non-linear dynamics of neuronal and behavioral processes directly from data, substituting themselves to “classic” *in silico* models (Fig. 2) as surrogate systems that can be interrogated without requiring additional experimentation—a practice known as dynamical system reconstruction, which remains a rapidly evolving field.^105^ In parallel, recent perspectives argue for reframing neural theory as a deep learning problem, using deep networks to capture and predict computations across scales, brain regions, and species.^106^ Together, these trends reflect a broader ambition to connect data-driven modeling and theoretical neuroscience through powerful machine learning frameworks. However, the promise of these methods comes with essential warnings. Neural systems are inherently stochastic, high-dimensional, and only partially observable; relevant variables may be missed, and multiple interacting timescales add further complexity. Moreover, while machine learning models can offer remarkable predictive power and a tight link to data, they are seldom constrained by biological realism. As Saxe *et al.*^106^ and Durstewitz *et al.*^105^ remind us, without careful design and critical interpretation, there is a risk that increasingly complex models will outpace our conceptual understanding. They suggest that, ideally, these models should be used as predictive models that generate falsifiable hypotheses and mechanistic insight, serving as bridges between detailed data and interpretable theories of brain function. This integration should span scales of description, from local circuits to behavior, while preserving the explanatory power that remains central to neuroscience.

## SUPPLEMENTARY MATERIAL

See the supplementary material for Table S1 for summarizing the studies selected for Secs. II and III. It includes the title(s) of the paragraph(s) for which the study was selected, a brief description of the *in silico* and related *in vitro* models, the main inherent results, and the *in silico* model repository link (when applicable).

## Data Availability

Data sharing is not applicable to this article as no new data were created or analyzed in this study.

## References

[1] R. Adolphs, “The unsolved problems of neuroscience,” Trends Cognit. Sci. 19(4), 173–175 (2015). 10.1016/j.tics.2015.01.007 25703689 PMC4574630

[2] H. Markram, “Seven challenges for neuroscience,” Funct. Neurol. 28(3), 145–151 (2013). 24139651 PMC3812747

[3] J. L. C. Lee, B. J. Everitt, and K. L. Thomas, “Independent cellular processes for hippocampal memory consolidation and reconsolidation,” Science 304(5672), 839–843 (2004). 10.1126/science.1095760 15073322

[4] A. Khadria, “Tools to measure membrane potential of neurons,” Biomed. J. 45(5), 749–762 (2022). 10.1016/j.bj.2022.05.007 35667642 PMC9661650

[5] M. E. J. Obien, K. Deligkaris, T. Bullmann, D. J. Bakkum, and U. Frey, “Revealing neuronal function through microelectrode array recordings,” Front. Neurosci. 8, 423 (2015). 10.3389/fnins.2014.00423 25610364 PMC4285113

[6] C. Magliaro and A. Ahluwalia, “To brain or not to brain organoids,” Front. Sci. 1, 1148873 (2023). 10.3389/fsci.2023.1148873

[7] R. A. McDougal, T. M. Morse, T. Carnevale, L. Marenco, R. Wang, M. Migliore, P. L. Miller, G. M. Shepherd, and M. L. Hines, “Twenty years of ModelDB and beyond: Building essential modeling tools for the future of neuroscience,” J. Comput. Neurosci. 42(1), 1–10 (2017). 10.1007/s10827-016-0623-7 27629590 PMC5279891

[8] S. Saray, C. A. Rossert, S. Appukuttan, R. Migliore, P. Vitale, C. A. Lupascu, L. L. Bologna, W. Van Geit, A. Romani, A. P. Davison, E. Muller, T. F. Freund, and S. Kali, “HippoUnit: A software tool for the automated testing and systematic comparison of detailed models of hippocampal neurons based on electrophysiological data,” PLoS Comput. Biol. 17(1), e1008114 (2021). 10.1371/journal.pcbi.1008114 33513130 PMC7875359

[9] K. Cranmer, J. Brehmer, and G. Louppe, “The frontier of simulation-based inference,” Proc. Natl. Acad. Sci. 117(48), 30055–30062 (2020). 10.1073/pnas.1912789117 32471948 PMC7720103

[10] W. Van Geit, E. De Schutter, and P. Achard, “Automated neuron model optimization techniques: A review,” Biol. Cybern. 99(4–5), 241–251 (2008). 10.1007/s00422-008-0257-6 19011918

[11] J. Van Pelt, P. S. Wolters, M. A. Corner, W. L. C. Rutten, and G. J. A. Ramakers, “Long-term characterization of firing dynamics of spontaneous bursts in cultured neural networks,” IEEE Trans. Biomed. Eng. 51(11), 2051–2062 (2004). 10.1109/TBME.2004.827936 15536907

[12] T. Opitz, A. D. De Lima, and T. Voigt, “Spontaneous development of synchronous oscillatory activity during maturation of cortical networks in vitro,” J. Neurophysiol. 88(5), 2196–2206 (2002). 10.1152/jn.00316.2002 12424261

[13] A. Arenas, A. Díaz-Guilera, J. Kurths, Y. Moreno, and C. Zhou, “Synchronization in complex networks,” Phys. Rep. 469(3), 93–153 (2008). 10.1016/j.physrep.2008.09.002

[14] H. Kamioka, E. Maeda, Y. Jimbo, H. P. C. Robinson, and A. Kawana, “Spontaneous periodic synchronized bursting during formation of mature patterns of connections in cortical cultures,” Neurosci. Lett. 206(2–3), 109–112 (1996). 10.1016/S0304-3940(96)12448-4 8710163

[15] T. A. Gritsun, J. Le Feber, J. Stegenga, and W. L. C. Rutten, “Network bursts in cortical cultures are best simulated using pacemaker neurons and adaptive synapses,” Biol. Cybern. 102(4), 293–310 (2010). 10.1007/s00422-010-0366-x 20157725

[16] S. Luccioli, E. Ben-Jacob, A. Barzilai, P. Bonifazi, and A. Torcini, “Clique of functional hubs orchestrates population bursts in developmentally regulated neural networks,” PLoS Comput. Biol. 10(9), e1003823 (2014). 10.1371/journal.pcbi.1003823 25255443 PMC4177675

[17] N. Maheswaranathan, S. Ferrari, A. M. J. VanDongen, and C. Henriquez, “Emergent bursting and synchrony in computer simulations of neuronal cultures,” Front. Comput. Neurosci. 6, 1–9 (2012). 10.3389/fncom.2012.00015 22514531 PMC3322356

[18] T. Masquelier and G. Deco, “Network bursting dynamics in excitatory cortical neuron cultures results from the combination of different adaptive mechanism,” PLoS One 8(10), e75824 (2013). 10.1371/journal.pone.0075824 24146781 PMC3795681

[19] E. Persi, D. Horn, V. Volman, R. Segev, and E. Ben-Jacob, “Modeling of synchronized bursting events: The importance of inhomogeneity,” Neural Comput. 16(12), 2577–2595 (2004). 10.1162/0899766042321823 15599972

[20] M.-L. Linne, J. Aćimović, A. Saudargiene, and T. Manninen, “Neuron–glia interactions and brain circuits,” in *Adv Exp Med Biol* (Springer, 2022), pp. 87–103. 10.1007/978-3-030-89439-9_4 35471536

[21] R. Kumar, Y.-T. Huang, C.-C. Chen, S.-F. Tzeng, and C.-K. Chan, “Astrocytic regulation of synchronous bursting in cortical cultures: From local to global,” Cereb. Cortex Commun. 1(1), tgaa053 (2020). 10.1093/texcom/tgaa053 34296118 PMC8153059

[22] H.-J. Jiang, J. Aćimović, T. Manninen, I. Ahokainen, J. Stapmanns, M. Lehtimäki, M. Diesmann, S. J. van Albada, H. E. Plesser, and M.-L. Linne, “Modeling neuron-astrocyte interactions in neural networks using distributed simulation,” BioRxiv:2024.11.11.622953 (2024). 10.1371/journal.pcbi.1013503 PMC12494295 40971948

[23] Y.-X. Li and J. Rinzel, “Equations for InsP_3_ receptor-mediated [Ca^2+^]_*i*_ oscillations derived from a detailed kinetic model: A Hodgkin-Huxley like formalism,” J. Theor. Biol. 166(4), 461–473 (1994). 10.1006/jtbi.1994.1041 8176949

[24] E. Dzyubenko, M. Fleischer, D. Manrique-Castano, M. Borbor, C. Kleinschnitz, A. Faissner, and D. M. Hermann, “Inhibitory control in neuronal networks relies on the extracellular matrix integrity,” Cell. Mol. Life Sci. 78(14), 5647–5663 (2021). 10.1007/s00018-021-03861-3 34128077 PMC8257544

[25] K. Lenk, B. Priwitzer, L. Ylä-Outinen, L. H. B. Tietz, S. Narkilahti, and J. A. K. Hyttinen, “Simulation of developing human neuronal cell networks,” Biomed. Eng. Online 15(1), 1–16 (2016). 10.1186/s12938-016-0226-6 PMC5006268 27576323

[26] J. Wen, M. Peitz, and O. Brüstle, “A defined human-specific platform for modeling neuronal network stimulation in vitro and in silico,” J. Neurosci. Methods 373, 109562 (2022). 10.1016/j.jneumeth.2022.109562 35292305

[27] N. Doorn, E. J. H. F. Voogd, M. R. Levers, M. J. A. M. van Putten, and M. Frega, “Breaking the burst: Unveiling mechanisms behind fragmented network bursts in patient-derived neurons,” Stem Cell Rep. 19(11), 1583–1597 (2024). 10.1016/j.stemcr.2024.09.001 PMC11589196 39366380

[28] K. Lenk, E. Satuvuori, J. Lallouette, A. Ladrón-de-Guevara, H. Berry, and J. A. K. Hyttinen, “A computational model of interactions between neuronal and astrocytic networks: The role of astrocytes in the stability of the neuronal firing rate,” Front. Comput. Neurosci. 13, 480462 (2020). 10.3389/fncom.2019.00092 PMC6987305 32038210

[29] T. Osaki, T. Duenki, S. Y. A. Chow, Y. Ikegami, R. Beaubois, T. Levi, N. Nakagawa-Tamagawa, Y. Hirano, and Y. Ikeuchi, “Complex activity and short-term plasticity of human cerebral organoids reciprocally connected with axons,” Nat. Commun. 15(1), 1–13 (2024). 10.1038/s41467-024-46787-7 38600094 PMC11006899

[30] J. Senk, B. Kriener, M. Djurfeldt, N. Voges, H. J. Jiang, L. Schüttler, G. Gramelsberger, M. Diesmann, H. E. Plesser, and S. J. van Albada, “Connectivity concepts in neuronal network modeling,” PLoS Comput. Biol. 18(9), e1010086 (2022). 10.1371/journal.pcbi.1010086 36074778 PMC9455883

[31] Y. Sato, H. Yamamoto, H. Kato, T. Tanii, S. Sato, and A. Hirano-Iwata, “Microfluidic cell engineering on high-density microelectrode arrays for assessing structure-function relationships in living neuronal networks,” Front. Neurosci. 16, 943310 (2023). 10.3389/fnins.2022.943310 36699522 PMC9868575

[32] K. Bansal, J. Nakuci, and S. F. Muldoon, “Personalized brain network models for assessing structure–function relationships,” Curr. Opin. Neurobiol. 52, 42–47 (2018). 10.1016/j.conb.2018.04.014 29704749

[33] M. Bonzanni and D. L. Kaplan, “On the prediction of neuronal microscale topology descriptors based on mesoscale recordings,” Eur. J. Neurosci. 54(6), 6147–6167 (2021). 10.1111/ejn.15417 34365680 PMC9209896

[34] J. G. Orlandi, J. Soriano, E. Alvarez-Lacalle, S. Teller, and J. Casademunt, “Noise focusing and the emergence of coherent activity in neuronal cultures,” Nat. Phys. 9(9), 582–590 (2013). 10.1038/nphys2686

[35] E. Tibau, A.-A. Ludl, S. Rudiger, J. G. Orlandi, and J. Soriano, “Neuronal spatial arrangement shapes effective connectivity traits of *in vitro* cortical networks,” IEEE Trans Network Sci. Eng. 7(1), 435–448 (2020). 10.1109/TNSE.2018.2862919

[36] J. M. Beggs and D. Plenz, “Neuronal avalanches in neocortical circuits,” J. Neurosci. 23(35), 11167–11177 (2003). 10.1523/JNEUROSCI.23-35-11167.2003 14657176 PMC6741045

[37] C. Tetzlaff, S. Okujeni, U. Egert, F. Wörgötter, and M. Butz, “Self-organized criticality in developing neuronal networks,” PLoS Comput. Biol. 6(12), e1001013 (2010). 10.1371/journal.pcbi.1001013 21152008 PMC2996321

[38] P. Massobrio, V. Pasquale, and S. Martinoia, “Self-organized criticality in cortical assemblies occurs in concurrent scale-free and small-world networks,” Sci. Rep. 5(1), 10578 (2015). 10.1038/srep10578 26030608 PMC4450754

[39] E. M. Izhikevich, “Simple model of spiking neurons,” IEEE Trans Neural Networks 14(6), 1569–1572 (2003). 10.1109/TNN.2003.820440 18244602

[40] T. T. Kanagasabapathi, P. Massobrio, R. A. Barone, M. Tedesco, S. Martinoia, W. J. Wadman, and M. M. J. Decré, “Functional connectivity and dynamics of cortical-thalamic networks co-cultured in a dual compartment device,” J. Neural Eng. 9(3), 036010 (2012). 10.1088/1741-2560/9/3/036010 22614532

[41] D. Poli, C. Magliaro, and A. Ahluwalia, “Experimental and computational methods for the study of cerebral organoids: A review,” Front. Neurosci. 13, 435423 (2019). 10.3389/fnins.2019.00162 PMC6411764 30890910

[42] S. Bosi, R. Rauti, J. Laishram, A. Turco, D. Lonardoni, T. Nieus, M. Prato, D. Scaini, and L. Ballerini, “From 2D to 3D: Novel nanostructured scaffolds to investigate signalling in reconstructed neuronal networks,” Sci. Rep. 5(1), 9562 (2015). 10.1038/srep09562 25910072 PMC5407555

[43] M. Bonzanni, K. M. Bockley, and D. L. Kaplan, “On the effect of neuronal spatial subsampling in small-world networks,” Eur. J. Neurosci. 53(2), 485–498 (2021). 10.1111/ejn.14937 32794296

[44] F. Callegari, M. Brofiga, and P. Massobrio, “Modeling the three-dimensional connectivity of *in vitro* cortical ensembles coupled to micro-electrode arrays,” PLoS Comput. Biol. 19(2), e1010825 (2023). 10.1371/journal.pcbi.1010825 36780570 PMC9956882

[45] M. A. Lancaster and J. A. Knoblich, “Organogenesisin a dish: Modeling development and disease using organoid technologies,” Science 345(6194), 2014 (1979). 10.1126/science.1247125 25035496

[46] J. Kim, B. K. Koo, and J. A. Knoblich, “Human organoids: Model systems for human biology and medicine,” Nat. Rev. Mol. Cell Biol. 21(10), 571–584 (2020). 10.1038/s41580-020-0259-3 32636524 PMC7339799

[47] J. Tan, S. Virtue, D. M. Norris, O. J. Conway, M. Yang, G. Bidault, C. Gribben, F. Lugtu, I. Kamzolas, J. R. Krycer, R. J. Mills, L. Liang, C. Pereira, M. Dale, A. S. Shun-Shion, H. J. M. Baird, J. A. Horscroft, A. P. Sowton, M. Ma, S. Carobbio, E. Petsalaki, A. J. Murray, D. C. Gershlick, J. A. Nathan, J. E. Hudson, L. Vallier, K. H. Fisher-Wellman, C. Frezza, A. Vidal-Puig, and D. J. Fazakerley, “Limited oxygen in standard cell culture alters metabolism and function of differentiated cells,” EMBO J. 43(11), 2127–2165 (2024). 10.1038/s44318-024-00084-7 38580776 PMC11148168

[48] T. L. Place, F. E. Domann, and A. J. Case, “Limitations of oxygen delivery to cells in culture: An underappreciated problem in basic and translational research,” Free Radical Biol. Med. 113, 311–322 (2017). 10.1016/j.freeradbiomed.2017.10.003 29032224 PMC5699948

[49] C. Magliaro, A. Rinaldo, and A. Ahluwalia, “Allometric scaling of physiologically-relevant organoids,” Sci. Rep. 9(1), 1–8 (2019). 10.1038/s41598-019-48347-2 31417119 PMC6695443

[50] Y. Wei, G. Ullah, J. Ingram, and S. J. Schiff, “Oxygen and seizure dynamics: II. Computational modeling,” J. Neurophysiol. 112(2), 213–223 (2014). 10.1152/jn.00541.2013 24671540 PMC4064403

[51] R. Fabbri, E. Botte, A. Ahluwalia, and C. Magliaro, “Digitoids: A novel computational platform for mimicking oxygen-dependent firing of neurons *in vitro*,” Front. Neuroinf. 19, 1549916 (2025). 10.3389/fninf.2025.1549916 PMC12259620 40665990

[52] I. Jaras, T. Harada, M. E. Orchard, P. E. Maldonado, and R. C. Vergara, “Extending the integrate-and-fire model to account for metabolic dependencies,” Eur. J. Neurosci. 54(4), 5249–5260 (2021). 10.1111/ejn.15326 34109698

[53] D. Calvetti, G. Capo Rangel, L. Gerardo Giorda, and E. Somersalo, “A computational model integrating brain electrophysiology and metabolism highlights the key role of extracellular potassium and oxygen,” J. Theor. Biol. 446, 238–258 (2018). 10.1016/j.jtbi.2018.02.029 29530764

[54] G. Capo-Rangel, L. Gerardo-Giorda, E. Somersalo, and D. Calvetti, “Metabolism plays a central role in the cortical spreading depression: Evidence from a mathematical model,” J. Theor. Biol. 486, 110093 (2020). 10.1016/j.jtbi.2019.110093 31778711

[55] G. Capo Rangel, J. Prezioso, L. Gerardo-Giorda, E. Somersalo, and D. Calvetti, “Brain energetics plays a key role in the coordination of electrophysiology, metabolism and hemodynamics: Evidence from an integrated computational model,” J. Theor. Biol. 478, 26–39 (2019). 10.1016/j.jtbi.2019.06.003 31175852

[56] A.-A. Ludl and J. Soriano, “Impact of physical obstacles on the structural and effective connectivity of *in silico* neuronal circuits,” Front. Comput. Neurosci. 14, 557191 (2020). 10.3389/fncom.2020.00077 PMC7488194 32982710

[57] C. A. Trujillo, J. W. Adams, P. D. Negraes, C. Carromeu, L. Tejwani, A. Acab, B. Tsuda, C. A. Thomas, N. Sodhi, K. M. Fichter, S. Romero, F. Zanella, T. J. Sejnowski, H. Ulrich, and A. R. Muotri, “Pharmacological reversal of synaptic and network pathology in human *MECP2*‐KO neurons and cortical organoids,” EMBO Mol. Med. 13(1), e12523 (2021). 10.15252/emmm.202012523 33501759 PMC7799367

[58] N. Doorn, E. J. H. van Hugte, U. Ciptasari, A. Mordelt, H. G. E. Meijer, D. Schubert, M. Frega, N. Nadif Kasri, and M. J. A. M. van Putten, “An *in silico* and *in vitro* human neuronal network model reveals cellular mechanisms beyond Na_V_1.1 underlying Dravet syndrome,” Stem Cell Rep. 18(8), 1686–1700 (2023). 10.1016/j.stemcr.2023.06.003 PMC10444571 37419110

[59] G. LeMasson, S. Przedborski, and L. F. Abbott, “A computational model of motor neuron degeneration,” Neuron 83(4), 975–988 (2014). 10.1016/j.neuron.2014.07.001 25088365 PMC4167823

[60] J. Alstott, M. Breakspear, P. Hagmann, L. Cammoun, and O. Sporns, “Modeling the impact of lesions in the human brain,” PLoS Comput. Biol. 5(6), e1000408 (2009). 10.1371/journal.pcbi.1000408 19521503 PMC2688028

[61] M. Kaiser, R. Martin, P. Andras, and M. P. Young, “Simulation of robustness against lesions of cortical networks,” Eur. J. Neurosci. 25(10), 3185–3192 (2007). 10.1111/j.1460-9568.2007.05574.x 17561832

[62] K. E. Joyce, S. Hayasaka, and P. J. Laurienti, “The human functional brain network demonstrates structural and dynamical resilience to targeted attack,” PLoS Comput. Biol. 9(1), e1002885 (2013). 10.1371/journal.pcbi.1002885 23358557 PMC3554573

[63] S. Faci-Lázaro, J. Soriano, and J. Gómez-Gardeñes, “Impact of targeted attack on the spontaneous activity in spatial and biologically-inspired neuronal networks,” Chaos 29(8), 083126 (2019). 10.1063/1.5099038 31472487

[64] E. Tibau, M. Valencia, and J. Soriano, “Identification of neuronal network properties from the spectral analysis of calcium imaging signals in neuronal cultures,” Front. Neural Circuits 7, 72027 (2013). 10.3389/fncir.2013.00199 PMC3866384 24385953

[65] S. V. Stasenko, A. E. Hramov, and V. B. Kazantsev, “Loss of neuron network coherence induced by virus-infected astrocytes: A model study,” Sci. Rep. 13(1), 6401 (2023). 10.1038/s41598-023-33622-0 37076526 PMC10115799

[66] M. V. Johnston, B. Mullaney, and M. E. Blue, “Neurobiology of Rett syndrome,” J. Child Neurol. 18(10), 688–692 (2003). 10.1177/08830738030180100501 14649550

[67] M. L. Gonzales and J. M. LaSalle, “The role of MeCP2 in brain development and neurodevelopmental disorders,” Curr. Psychiatry Rep. 12(2), 127–134 (2010). 10.1007/s11920-010-0097-7 20425298 PMC2847695

[68] M. V. C. Nguyen, F. Du, C. A. Felice, X. Shan, A. Nigam, G. Mandel, J. K. Robinson, and N. Ballas, “MeCP2 is critical for maintaining mature neuronal networks and global brain anatomy during late stages of postnatal brain development and in the mature adult brain,” J. Neurosci. 32(29), 10021–10034 (2012). 10.1523/JNEUROSCI.1316-12.2012 22815516 PMC3461266

[69] R. S. F. Mok, W. Zhang, T. I. Sheikh, K. Pradeepan, I. R. Fernandes, L. C. DeJong, G. Benigno, M. R. Hildebrandt, M. Mufteev, D. C. Rodrigues, W. Wei, A. Piekna, J. Liu, A. R. Muotri, J. B. Vincent, L. Muller, J. Martinez-Trujillo, M. W. Salter, and J. Ellis, “Wide spectrum of neuronal and network phenotypes in human stem cell-derived excitatory neurons with Rett syndrome-associated MECP2 mutations,” Transl. Psychiatry 12(1), 450 (2022). 10.1038/s41398-022-02216-1 36253345 PMC9576700

[70] R. Takayama, T. Fujiwara, H. Shigematsu, K. Imai, Y. Takahashi, K. Yamakawa, and Y. Inoue, “Long-term course of Dravet syndrome: A study from an epilepsy center in Japan,” Epilepsia 55(4), 528–538 (2014). 10.1111/epi.12532 24502503

[71] C. Dravet, “The core Dravet syndrome phenotype,” Epilepsia 52(Suppl. 2), 3–9 (2011). 10.1111/j.1528-1167.2011.02994.x 21463272

[72] F. Ragona, “Cognitive development in children with Dravet syndrome,” Epilepsia 52(Suppl. 2), 39–43 (2011). 10.1111/j.1528-1167.2011.03000.x 21463278

[73] N. Doorn, M. J. A. M. van Putten, and M. Frega, “Automated inference of disease mechanisms in patient-hiPSC-derived neuronal networks,” Commun. Biol. 8(1), 768 (2025). 10.1038/s42003-025-08209-2 40394269 PMC12092834

[74] G. T. Kress, F. Chan, C. A. Garcia, and W. S. Merrifield, “Utilizing machine learning algorithms to predict subject genetic mutation class from in silico models of neuronal networks,” BMC Med. Inf. Decis. Making 22(1), 290 (2022). 10.1186/s12911-022-02038-7 PMC9647930 36352381

[75] N. H. Lam, T. Borduqui, J. Hallak, A. Roque, A. Anticevic, J. H. Krystal, X. J. Wang, and J. D. Murray, “Effects of altered excitation-inhibition balance on decision making in a cortical circuit model,” J. Neurosci. 42(6), 1035–1053 (2022). 10.1523/JNEUROSCI.1371-20.2021 34887320 PMC8824494

[76] B. Mossink, J. R. van Rhijn, S. Wang, K. Linda, M. R. Vitale, J. E. M. Zöller, E. J. H. van Hugte, J. Bak, A. H. A. Verboven, M. Selten, M. Negwer, B. L. Latour, I. van der Werf, J. M. Keller, T. M. Klein Gunnewiek, C. Schoenmaker, A. Oudakker, A. Anania, S. Jansen, K. P. Lesch, M. Frega, H. van Bokhoven, D. Schubert, and N. Nadif Kasri, “Cadherin-13 is a critical regulator of GABAergic modulation in human stem-cell-derived neuronal networks,” Mol. Psychiatry 27(1), 1–18 (2022). 10.1038/s41380-021-01117-x 33972691 PMC8960401

[77] E. Dzyubenko, G. Juckel, and A. Faissner, “The antipsychotic drugs olanzapine and haloperidol modify network connectivity and spontaneous activity of neural networks *in vitro*,” Sci. Rep. 7(1), 11609 (2017). 10.1038/s41598-017-11944-0 28912551 PMC5599625

[78] J. K. Liu and Z. S. She, “A spike-timing pattern based neural network model for the study of memory dynamics,” PLoS One 4(7), e6247 (2009). 10.1371/journal.pone.0006247 19629179 PMC2710501

[79] T. Kim, R. A. Capps, K. C. Hamade, W. H. Barnett, D. I. Todorov, E. M. Latash, S. N. Markin, I. A. Rybak, and Y. I. Molkov, “The functional role of striatal cholinergic interneurons in reinforcement learning from computational perspective,” Front. Neural Circuits 13, 10 (2019). 10.3389/fncir.2019.00010 30846930 PMC6393383

[80] H. Ju, M. R. Dranias, G. Banumurthy, and A. M. J. VanDongen, “Spatiotemporal memory is an intrinsic property of networks of dissociated cortical neurons,” J. Neurosci. 35(9), 4040–4051 (2015). 10.1523/JNEUROSCI.3793-14.2015 25740531 PMC6605577

[81] W. Van Geit, M. Gevaert, G. Chindemi, C. Rössert, J.-D. Courcol, E. B. Muller, F. Schürmann, I. Segev, and H. Markram, “BluePyOpt: Leveraging open source software and cloud infrastructure to optimise model parameters in neuroscience,” Front. Neuroinf. 10, 195490 (2016). 10.3389/fninf.2016.00017 PMC4896051 27375471

[82] J.-M. Lueckmann, J. Boelts, D. S. Greenberg, P. J. Gonçalves, and J. H. Macke, “Benchmarking simulation-based inference,” in *International Conference on Artificial Intelligence and Statistics* (PMLR, 2021), pp. 343–351.

[83] J.-M. Lueckmann, P. J. Goncalves, G. Bassetto, K. Öcal, M. Nonnenmacher, and J. H. Macke, “Flexible statistical inference for mechanistic models of neural dynamics,” in Advances in Neural Information Process Systems 30 (2017).

[84] P. J. Gonçalves, J.-M. Lueckmann, M. Deistler, M. Nonnenmacher, K. Öcal, G. Bassetto, C. Chintaluri, W. F. Podlaski, S. A. Haddad, T. P. Vogels, D. S. Greenberg, and J. H. Macke, “Training deep neural density estimators to identify mechanistic models of neural dynamics,” eLife 9, 1–46 (2020). 10.7554/eLife.56261 PMC7581433 32940606

[85] D. S. Greenberg, M. Nonnenmacher, and J. H. Macke, “Automatic posterior transformation for likelihood-free inference,” in Proceedings of the 36th International Conference on Machine Learning (2019), pp. 2404–2414, PMLR. https://proceedings.mlr.press/v97/greenberg19a.html

[86] G. Papamakarios, D. C. Sterratt, and I. Murray, “Sequential neural likelihood: Fast likelihood-free inference with autoregressive flows,” in Proceedings of the Twenty-Second International Conference on Artificial Intelligence and Statistics (2019), pp. 837–848. PMLR. https://proceedings.mlr.press/v89/papamakarios19a.html

[87] J. Hermans, V. Begy, and G. Louppe, “Likelihood-free MCMC with amortized approximate ratio estimators,” in Proceedings of the 37th International Conference on Machine Learning (2020), pp. 4239–4248. PMLR. https://proceedings.mlr.press/v119/hermans20a.html

[88] S. R. Bittner, A. Palmigiano, A. T. Piet, C. A. Duan, C. D. Brody, K. D. Miller, and J. Cunningham, “Interrogating theoretical models of neural computation with emergent property inference,” eLife 10, e56265 (2021). 10.7554/eLife.56265 34323690 PMC8321557

[89] S. Talts, M. Betancourt, D. Simpson, A. Vehtari, and A. Gelman, “Validating Bayesian inference algorithms with simulation-based calibration,” arXiv:1804.06788v2 (2020).

[90] A. Tejero-Cantero, J. Boelts, M. Deistler, J.-M. Lueckmann, C. Durkan, P. Gonçalves, D. Greenberg, and J. Macke, “sbi: A toolkit for simulation-based inference,” J. Open Source Software 5(52), 2505 (2020). 10.21105/joss.02505

[91] H. Markram, “The human brain project,” Sci. Am. 306(6), 50–55 (2012). 10.1038/scientificamerican0612-50 22649994

[92] S. Dura-Bernal, B. A. Suter, P. Gleeson, M. Cantarelli, A. Quintana, F. Rodriguez, D. J. Kedziora, G. L. Chadderdon, C. C. Kerr, S. A. Neymotin, R. A. McDougal, M. Hines, G. M. G. Shepherd, and W. W. Lytton, “NetpyNE, a tool for data-driven multiscale modeling of brain circuits,” eLife 8, e44494 (2019). 10.7554/eLife.44494 31025934 PMC6534378

[93] R. De Schepper, A. Geminiani, S. Masoli, M. F. Rizza, A. Antonietti, C. Casellato, and E. D'Angelo, “Model simulations unveil the structure-function-dynamics relationship of the cerebellar cortical microcircuit,” Commun. Biol. 5(1), 1–19 (2022). 10.1038/s42003-022-04213-y 36376444 PMC9663576

[94] M. L. Hines and N. T. Carnevale, “The NEURON simulation environment,” Neural Comput. 9(6), 1179–1209 (1997). 10.1162/neco.1997.9.6.1179 9248061

[95] J. M. Bower and D. Beeman, *The Book of Genesis* (Springer, New York, 1998).

[96] M.-O. Gewaltig and M. Diesmann, “NEST (NEural Simulation Tool),” Scholarpedia 2(4), 1430 (2007). 10.4249/scholarpedia.1430

[97] M. Stimberg, R. Brette, and D. F. M. Goodman, “Brian 2, an intuitive and efficient neural simulator,” eLife 8, e47314 (2019). 10.7554/eLife.47314 31429824 PMC6786860

[98] A. P. Davison, D. Brüderle, J. Eppler, J. Kremkow, E. Muller, D. Pecevski, L. Perrinet, and P. Yger, “PyNN: A common interface for neuronal network simulators,” Front. Neuroinf. 2, 388 (2008). 10.3389/neuro.11.011.2008 PMC2634533 19194529

[99] M. D. Wilkinson, M. Dumontier, I. J. Aalbersberg, G. Appleton, M. Axton, A. Baak, N. Blomberg, J. W. Boiten, L. B. da Silva Santos, P. E. Bourne, J. Bouwman, A. J. Brookes, T. Clark, M. Crosas, I. Dillo, O. Dumon, S. Edmunds, C. T. Evelo, R. Finkers, A. Gonzalez-Beltran, A. J. G. Gray, P. Groth, C. Goble, J. S. Grethe, J. Heringa, P. A. C't Hoen, R. Hooft, T. Kuhn, R. Kok, J. Kok, S. J. Lusher, M. E. Martone, A. Mons, A. L. Packer, B. Persson, P. Rocca-Serra, M. Roos, R. van Schaik, S. A. Sansone, E. Schultes, T. Sengstag, T. Slater, G. Strawn, M. A. Swertz, M. Thompson, J. Van Der Lei, E. Van Mulligen, J. Velterop, A. Waagmeester, P. Wittenburg, K. Wolstencroft, J. Zhao, and B. Mons, “The FAIR guiding principles for scientific data management and stewardship,” Sci. Data 3(1), 160018 (2016). 10.1038/sdata.2016.18 PMC4792175 26978244

[100] J. Cornell, S. Salinas, H.-Y. Huang, and M. Zhou, “Microglia regulation of synaptic plasticity and learning and memory,” Neural Regener. Res. 17(4), 705 (2022). 10.4103/1673-5374.322423 PMC8530121 34472455

[101] M. Munyeshyaka and R. D. Fields, “Oligodendroglia are emerging players in several forms of learning and memory,” Commun. Biol. 5(1), 1148 (2022). 10.1038/s42003-022-04116-y 36309567 PMC9617857

[102] C. Anand, P. D. Maia, J. Torok, C. Mezias, and A. Raj, “The effects of microglia on tauopathy progression can be quantified using Nexopathy *in silico* (Nex*is*) models,” Sci. Rep. 12(1), 21170 (2022). 10.1038/s41598-022-25131-3 36477076 PMC9729195

[103] A. Foster‐Powell, A. Rostami‐Hodjegan, G. Meno‐Tetang, D. E. Mager, and K. Ogungbenro, “Mathematical modeling of neuroinflammation in neurodegenerative diseases,” CPT: Pharmacometrics Syst. Pharmacol. 1–15 (2025). 10.1002/psp4.70064 PMC12706407 40801430

[104] P. J. Gebicke-Haerter, “The computational power of the human brain,” Front. Cell Neurosci. 17, 1220030 (2023). 10.3389/fncel.2023.1220030 37608987 PMC10441807

[105] D. Durstewitz, G. Koppe, and M. I. Thurm, “Reconstructing computational system dynamics from neural data with recurrent neural networks,” Nat. Rev. Neurosci. 24(11), 693–710 (2023). 10.1038/s41583-023-00740-7 37794121

[106] A. Saxe, S. Nelli, and C. Summerfield, “If deep learning is the answer, what is the question?,” Nat. Rev. Neurosci. 22(1), 55–67 (2021). 10.1038/s41583-020-00395-8 33199854

